# Time-Domain Analysis of Low- and High-Frequency Near-Infrared Spectroscopy Sensor Technologies for Characterization of Cerebral Pressure–Flow and Oxygen Delivery Physiology: A Prospective Observational Study

**DOI:** 10.3390/s25175391

**Published:** 2025-09-01

**Authors:** Amanjyot Singh Sainbhi, Nuray Vakitbilir, Tobias Bergmann, Kevin Y. Stein, Rakibul Hasan, Noah Silvaggio, Mansoor Hayat, Jaewoong Moon, Frederick A. Zeiler

**Affiliations:** 1 Department of Biomedical Engineering, Price Faculty of Engineering, University of Manitoba, Winnipeg, MB R3T 5V6, Canada; vakitbir@myumanitoba.ca (N.V.); bergmant@myumanitoba.ca (T.B.); steink34@myumanitoba.ca (K.Y.S.); hasanr2@myumanitoba.ca (R.H.); frederick.zeiler@umanitoba.ca (F.A.Z.); 2 Undergraduate Medicine, Rady Faculty of Health Sciences, University of Manitoba, Winnipeg, MB R3T 3P5, Canada; 3 Department of Human Anatomy and Cell Science, Rady Faculty of Health Sciences, University of Manitoba, Winnipeg, MB R3T 0J9, Canada; silvaggn@myumanitoba.ca; 4 Section of Neurosurgery, Department of Surgery, Rady Faculty of Health Sciences, University of Manitoba, Winnipeg, MB R3T 1R9, Canada; mansoor.hayat@umanitoba.ca (M.H.); jaewoong.moon@umanitoba.ca (J.M.); 5 Pan Am Clinic Foundation, Winnipeg, MB R3M 3E4, Canada; 6 Department of Clinical Neurosciences, Karolinska Institutet, 171 77 Stockholm, Sweden; 7 Division of Anaesthesia, Department of Medicine, Addenbrooke’s Hospital, University of Cambridge, Cambridge CB2 0QQ, UK

**Keywords:** ARIMA structure, cerebral oximetry index, regional cerebral oxygen saturation, time domain, time series

## Abstract

Cerebrovascular reactivity, cerebral autoregulation (CA), and oxygen delivery can be measured continuously and in a non-invasive fashion using cerebral near-infrared spectroscopy (NIRS). Although the literature is limited surrounding the difference between signals acquired and derived from low (<100 Hz) and high sampling rates (≥100 Hz). As part of a prospective observational study, we preliminarily explored and assessed the difference in the information provided by two NIRS systems using regional cerebral oxygen saturation and cerebral oximetry index signals at low and high sampling rates. The raw data in two frequencies (down-sampled to 1 Hz using the mean and up-sampled to 250 Hz) were decimated to focus on slow-wave vasogenic fluctuations associated with CA. Then, the data were analyzed using various statistical methods such as the absolute signal difference, Pearson correlation, Bland–Altman agreement, Cross-correlation function, optimal time-series autocorrelative structure, time-series impulse response function, and Granger causality relationships. The results of the various statistical analyses indicated that the signals obtained using high-frequency NIRS were different from signals obtained from low-frequency NIRS of the same cerebral region. Hence, high-frequency NIRS systems may possibly contain better signal features compared to NIRS systems with low sampling rates, but further work is required to assess high-frequency NIRS in other healthy and cranial trauma populations.

## 1. Introduction

Cerebral physiology encompasses the brain’s functional mechanisms and operations, including cerebral pressure–flow and oxygen delivery physiology (such as cerebral blood flow [CBF], cerebral blood volume [CBv], arterial blood pressure [ABP], intracranial pressure [ICP], cerebral perfusion pressure [CPP], and intricate regulatory mechanisms). Cerebral autoregulation (CA) is a physiologic process that regulates CBF to ensure optimal brain function by maintaining a relatively constant CBF over a wide range of systemic ABP [1,2]. A broader term for CA is cerebrovascular reactivity (CVR), which is the mechanism behind the maintenance of the constant CBF by constricting and dilating cerebral blood vessels [1,2]. With the brain exposed to pressure-passive flow states, hypoperfusion or hyperperfusion can occur due to either low or high CPP/ABP, respectively, outside the range of naturally maintainable ABP [1,2,3]. The impairment of CA has been documented in both healthy and pathological states [4,5,6,7,8,9,10], and it plays a significant role in unfavorable long-term outcomes in various neuropathological conditions [6,7,9,11,12,13].

With the use of CVR metrics, which are surrogate measures of CA, we can obtain indirect measurements of CA at the bedside [4,14,15] and assess the association between slow-wave (i.e., 0.05–0.005 Hz) vasogenic fluctuations [4,15,16,17] in ABP and a surrogate for CBv. Although the most established invasive method for continuous time domain CVR assessment is ICP-based [18,19,20], the non-invasive monitoring of various aspects of cerebral physiology can be achieved using near-infrared spectroscopy (NIRS) systems that measure relative oxygenated and deoxygenated hemoglobin in the brain tissue based on absorption of near-infrared (NIR) light using the Modified Beer–Lambert Law [21,22] in the specific range of NIR wavelengths from 650 nm to 950 nm [22,23], and NIRS-based CVR indices have been described as a substitute for ICP-based indices [24,25,26,27,28,29,30]. A surrogate for pulsatile CBv is the NIRS-based regional cerebral oxygen saturation (rSO_2_) signal, which refers to the local cerebral oxygen content [26,27,31,32,33,34,35,36]. By correlating rSO_2_ with CPP/ABP using moving Pearson correlation of five minute data updated every 10 s, the CVR metric known as the cerebral oximetry index (COx/COx-a, respectively) [22,26,29,31,32,33,34,35,37,38,39] has been shown to measure aspects of the lower limit of autoregulation in pre-clinical literature [24,26], similar to “gold standard” invasive ICP-based CVR metrics calculated in the same manner. The advantages of NIRS-based signals over ICP-based signals are that they have the potential to be applied in different populations due to their non-invasive nature, and they can be used in a multi-channel capacity, which overcomes the spatial resolution issue associated with ICP-based signals.

At present, the literature surrounding the difference between the signals acquired and derived from low sampling rate (<100 Hz) cerebral NIRS systems, often using commercial systems in healthcare settings, and high sampling rate (≥100 Hz) cerebral NIRS systems, which use research-grade cerebral NIRS systems, is limited. The 100 Hz division of low versus high sampling rates was used because it satisfies the Nyquist criterion for the recommended frequency of ≥50 Hz to accurately capture valid heart rate signals, as defined by Burma and colleagues, and it meets their ≥90 Hz frequency requirement to obtain valid heart rate variability metrics derived from pulsatile waveforms [40]. In the current literature, discerning hemispheric regional disparity in both health and disease produced varying results in a previously conducted scoping review by our group [41]. Therefore, a recent study was conducted by our group, which found that a commercially available cerebral NIRS system with a low sampling rate (~1 Hz) may not be able to detect hemispheric regional disparity using continuously measured COx/COx-a in a cranial trauma population [42]. It remains unknown if the time-series structure changes based on the amount of NIRS data collected using low versus high sampling rates. The goal of this prospective observational study is to explore and assess the difference in the information provided by low and high sampling rates using a commercial-grade NIRS system and a research-grade functional NIRS (fNIRS) system, respectively.

## 2. Materials and Methods

### 2.1. Study Design and Human Population

This study was conducted as a registered prospective observational study (ClinicalTrials.gov ID: NCT05433129) at the University of Manitoba Multi-omic Analytics and Integrative Neuroinformatics in the HUman Brain (MAIN-HUB) Lab [43,44]. Currently, this study has completed the first of two phases of data collection, and this manuscript represents the first small aspect of analysis addressing the study’s first objective, with data analysis conducted similarly to previous works from our group [42,45,46,47]. Presently, there is a lack of available summarized findings related to this registered study, as previously referenced, due to its multi-phase nature. Further analyses related to the first objective are in the works, while data collection for the study’s second objective is ongoing. The detailed study protocol was provided in our previously published manuscript [43]. In short, all healthy volunteers (HVAs) were 18 years or older and had no history of neurological or cardiovascular conditions. The prospective observational study consisted of simultaneous single-channel low-frequency and multi-channel high-frequency cerebral NIRS recordings during baseline and various perturbation testing in a block-trial fashion. Perturbations included assessments of vascular chemo-reactivity through breath-holding challenges (with end-tidal CO_2_ monitoring), orthostatic position changes, and neurovascular coupling assessments through formal cognitive testing using the Clinical Toolkit test battery in the Automated Neuropsychological Assessment Metrics (ANAM) test system software (Version 4.5.0.6, Vista Life Sciences, Parker, CO, USA). These perturbations were employed in this prospective observational study to disturb the CA in healthy volunteers to measure various NIRS-based CVR indices at each brain lobe and hemisphere, and it can be used as a “gold standard” for future comparisons to cranial trauma population data recorded using similar higher-frequency NIRS systems. All recordings occurred continuously over approximately 1.5 h for each subject.

### 2.2. Ethical Considerations

Data were collected following full approval by the University of Manitoba Health Research Ethics Board (B2022:051) and the Shared Health/Health Sciences Centre Research Impact Committee (SH2022-210). Additionally, the study is registered on ClinicalTrials.gov (ID: NCT05433129).

### 2.3. Data Collection

Leveraging a custom multi-channel fNIRS system OxyMon Mk III (Artinis Medical Systems, Elst, The Netherlands), four types of relative hemoglobin signals (oxyhemoglobin [HbO], deoxyhemoglobin [HHb], total hemoglobin [tHb], and the difference between HbO and HHb [HbDiff]) were recorded at high frequency (250 Hz), with the differential pathlength factor set at 6 for brain tissue monitoring, using short (transmitter and receiver optode distance of 10 mm) and normal channels (transmitter and receiver optode distance of 30 mm), and using the fNIRS cap to hold the optodes on a human head [44]. Although eight channels were used to assess each of the four brain lobes (frontal, parietal, temporal, and occipital) on both hemispheres of the brain with perturbations targeting the whole system, only the signals from the frontal right channel, superficial to the right frontal lobe, were leveraged for the study to facilitate comparison of data streams from the OxyMon system (i.e., research-grade high-frequency) to the INVOS system (i.e., commercial-grade low-frequency) in the same brain region (i.e., right frontal). The short channel signal was subtracted from the normal channel signal to eliminate extracranial signal contamination and produce the differenced HbO, HHb, tHb, and HbDiff signals. The median and IQR of the relative HHb and tHb related to the right frontal brain region are given in File S1a. The frontal right rSO_2_ (rSO_2__Oxymon) signal without extracranial signal contamination was derived by expressing the differenced HHb as a percentage of the differenced tHb (i.e., ${rSO}_{2}=\frac{HHb}{tHb}*100$).

The ABP was non-invasively collected at 100 Hz using the Finapres Nova finger cuff (Finapres Medical Systems, Enschede, The Netherlands). Using a commercial-grade NIRS regional oximetry monitoring system (INVOS 5100C or 7100; Medtronic, Minneapolis, MN, USA), rSO_2_ was measured using a disposable adhesive pad on the right forehead (consisting of two transmitters and one receiver optode positioned at 180 degrees, with near and far transmitter–receiver distances of 30 mm and 40 mm, respectively), superficial to the right frontal lobe (rSO_2__Invos). This device uses a proprietary algorithm to only output the calculated rSO_2_ value from relative amounts of HHb and tHb in a spatially resolved manner to avoid superficial extracranial tissue contamination, but the exact derivation of the rSO_2__Invos value from the commercial system is unknown. The INVOS and OxyMon NIRS channels were spaced over the right frontal region in a manner to avoid cross-contamination between the devices while simultaneously recording.

**Figure 1:**
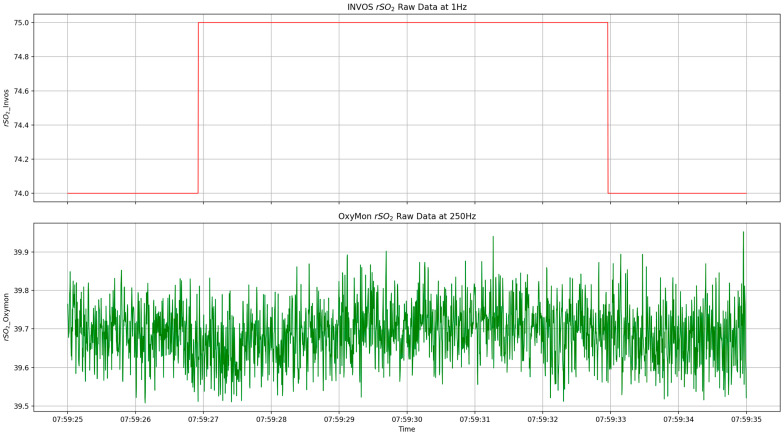
The figure shows a 10-s portion of a subject’s front right rSO_2_ signals from INVOS and OxyMon NIRS devices with a sampling frequency of 1 Hz and 250 Hz, respectively. This plot depicts the amount of data missing when rSO_2_ is sampled at a low frequency versus a high frequency and includes full waveform data. rSO_2_, regional cerebral oxygen saturation.

All physiological data were recorded and digitized in high-frequency resolution (up to 250 Hz where available) using a custom recording Python module and analog-to-digital signal converters (DT9804 or DT9826, Data Translation, Marlboro, MA, USA) where needed. The data quality comparison was performed on data in two frequencies (1 Hz and 250 Hz), and all physiological data were either down-sampled to 1 Hz using the mean or up-sampled to 250 Hz. Figure 1 depicts a 10-s portion of a subject’s rSO_2_ signal using low-frequency and high-frequency NIRS systems to show how much signal information can be lost due to low sampling frequency.

### 2.4. Physiologic Data Cleaning and Processing

The recorded high-resolution physiological data were cleaned and processed afterwards in Python 3.13.3. The signal artifacts related to equipment checks and adjustments, equipment malfunction, and degradation of signal quality were removed from the data streams using erroneous label events noted during the recording. For each patient, the ABP, rSO_2__OxyMon, and rSO_2__Invos raw signals were decimated using non-overlapping moving average filters of 10-s duration to focus on the slow-wave vasogenic fluctuations associated with CA [16,17,24]. Since the decimation data are sampled at 0.1 Hz, due to the applied non-overlapping moving average filter, this eliminates high-frequency noise from pulse and respiratory frequencies and facilitates the detection of low-frequency oscillations and transient events under 0.05 Hz [16,24], according to the Nyquist theorem. The moving Pearson correlation coefficients were calculated using 30 consecutive 10-s mean windows (i.e., five minutes of data), updated every 10 s, to derive the following rSO_2_-based CVR index using ABP for both NIRS devices [24,48]: right cerebral oximetry index with ABP for the OxyMon system (COx-a_Oxymon—correlation between rSO_2__Oxymon and ABP) and right cerebral oximetry index with ABP for the INVOS system (COx-a_Invos—correlation between rSO_2__Invos and ABP). This NIRS-based CVR index calculation is consistent with practices in neurocritical care monitoring and has been shown to correlate with ICP-based CVR indices [26,29,49]. These CVR indices have a range from −1 to +1, since they were computed using Pearson correlation coefficients, with higher values (closer to +1) indicating an impaired CVR and values below the range of 0 to +0.4 indicating an intact CVR [24,50].

### 2.5. Statistical Data Analysis

All the statistical analyses were performed in Python with alpha (α) set at 0.05 for statistical significance. General data distributions were summarized using median, interquartile range (IQR), number, and percentage, where appropriate. The overall analysis of the rSO_2_ and COx-a data quality comparison encompassed the following:

1. Absolute differences between each of the rSO_2_ and COx-a signals obtained using OxyMon and INVOS systems (raw and 10-s decimated data);
2. Pearson correlation, Bland–Altman agreement, and cross-correlation function comparison between each of the rSO_2_ and COx-a signals obtained using OxyMon and INVOS systems (raw and 10-s decimated data);
3. Optimal time-series autocorrelative structure differences for rSO_2_ and COx-a signals between OxyMon and INVOS systems (1st order differenced data at 1 Hz and 250 Hz);
4. Time-series impulse response function differences for ABP on rSO_2_ relationships between OxyMon and INVOS systems (1st order differenced at 1 Hz and 250 Hz);
5. Difference in Granger causality relationships between ABP and rSO_2_ using OxyMon and INVOS systems (1st order differenced data at 1Hz and 250 Hz).
6. In the sub-sections to follow, the individual methods will be briefly covered.

#### 2.5.1. Absolute Signal Difference—Raw and 10-s Decimated Data

The signal disparity of right frontal rSO_2_ and COx-a signals was determined in individual patients between signals obtained from both NIRS systems using raw and 10-s decimated data at 1 Hz and 250 Hz. With a custom script developed in Python, the absolute value of the signal difference was calculated for each subject using the right frontal signal obtained from the OxyMon and INVOS systems to determine the absolute signal difference (ASD). The ASD is able to provide a quantifiable magnitude of difference for a signal between the NIRS systems. We derived the median absolute deviation (MAD) of the ASDs for the entire population and in subgroup analysis by finding the median of the absolute deviation of each ASD value from the median of the ASD.

#### 2.5.2. Pearson Correlation Analysis—Raw and 10-s Decimated Data

Pearson correlation analysis was performed on signals obtained using each NIRS system to check for their correlation in each signal for a subject using raw and 10-s decimated data at 1 Hz and 250 Hz. The Pearson correlation coefficient (*r*) and *p*-value were obtained, along with their median and IQR values in both frequencies. The value of *r* indicates the strength of the correlation, and the *p*-value signifies whether the result was significant, with α set at 0.05 for statistical significance.

#### 2.5.3. Bland–Altman Agreement Analysis—Raw and 10-s Decimated Data

Bland–Altman agreement analysis was performed to check the agreement between each signal obtained using OxyMon and INVOS NIRS systems for a subject using raw and 10-s decimated data at 1 Hz and 250 Hz. The bias, limit of agreement (LoA), spread of the LoA, bias as a proportion of the LoA spread (relative bias), and the regressed slope and intercept were obtained along with their median and IQR values in both frequencies. The bias is the mean difference, which indicates the magnitude of the systemic difference. The LoA is the mean difference with ±1.96 standard deviations of difference. The spread of the LoA provides information about the agreement. The relative bias helps determine the magnitude of bias relative to the global changeability of the differences, and the regression equation provides information about the underestimation or overestimation.

#### 2.5.4. Cross-Correlation Function Analysis—Raw and 10-s Decimated Data

Cross-correlation function analysis was performed to measure the similarity between each signal obtained using OxyMon and INVOS NIRS systems with raw and 10-s decimated data at 1 Hz and 250 Hz. The correlation values for positive and negative lags provide information on how much the first time-series is related to the other at the lags, and the lag where the correlation value peaks provide information on the lead–lag relationship of those signals.

#### 2.5.5. Optimal Time-Series Structures of rSO_2_ and COx-a Signals—1 Hz and 250 Hz Sampled Data

General methods for optimal time-series autoregressive integrative moving average (ARIMA) structures can be found in File S1b,c and our previous work on the subject [42,47,51]. In brief, the data stationarity and optimal autoregressive order (p-order) and moving average order (q-order) were determined for rSO_2_ and COx-a signal sources in both frequencies using standard Box–Jenkins methodologies while the integrative order (d-order) was held at 1 [52,53,54]. Stationarity analysis was performed for each physiologic signal at an individual level using augmented Dickey–Fuller (ADF) and Kwiatkowski–Phillips–Schmidt–Shin (KPSS) tests [54]. Optimal ARIMA model orders were determined using the lowest Akaike information criterion (AIC) values for the derived ARIMA models. A median optimal ARIMA model order was determined for each signal in both frequencies, along with the IQR of the optimal ARIMA model order across the population.

#### 2.5.6. Time-Series Impulse Response Function (IRF) for ABP and rSO_2_—First-Order Differenced Data

A general description of vector ARIMA (VARIMA) models can be found in File S1d and our previous work in the area [42,45]. In brief, VARIMA models were derived to evaluate signal differences between both NIRS systems for the multivariate relationship between ABP and rSO_2_ (i.e., the constituents of NIRS-based CVR index COx-a). The p-order for the VARIMA model was calculated by taking the product of the optimal ARIMA p-orders of the two signals being evaluated, while the VARIMA q-order was calculated by adding the optimal ARIMA q-orders of the two signals being evaluated, as suggested in past literature [53]. IRF coefficients of the VARIMA model were generated using the optimal VARIMA model for the normalized first-order differenced data in both frequencies. The responsiveness of various models was checked to see if it was greater than an absolute value of 0.001. This threshold was chosen to look at a change in the normalized response of at least 0.1% in the majority of five lags after lag 10 (lags 11 to 15). In each population, the number of patients showing a greater response than 0.1% was counted for each directionality relationship between two signals. The VARIMA IRF analysis was used to compare the cerebral physiologic relationships of rSO_2_ from both NIRS devices with ABP to evaluate the dynamic temporal influence of a signal on another after a lag of 10 using bivariate VARIMA models.

#### 2.5.7. Differences in Granger Causality Relationships Between ABP and rSO_2_—First-Order Differenced Data

To evaluate the interdependence between ABP and rSO_2_ (i.e., the constituents of NIRS-based CA/CVR index COx-a) and any signal disparities, we leveraged Granger causality testing. Granger causality testing was used to assess the ability of one signal to predict another signal beyond the ability of the signal to predict itself. Using the first-order-differenced data in both frequencies, the responses of Granger causality tests, both F-test statistical values and *p*-values [55], were recorded. The identification of reciprocal influences between two signals was assessed from these responses, and the cerebral physiologic relationships between rSO_2_ from both NIRS devices with ABP were compared to identify the difference in the directional predictability using past values.

#### 2.5.8. Subgroup Analysis

All the above aspects of analysis for the signal disparity between both NIRS modalities for rSO_2_ and COx-a signals using the full recording (baseline and perturbation data) were also conducted in various subgroups based on perturbation tests (neurovascular coupling, orthostatic challenge, and vascular chemo-reactivity), along with the baseline.

## 3. Results

### 3.1. Population Demographics

A total of 50 HVAs were included in this study. The median recording duration was 89.5 min (IQR: 85.7–95.3 min). Table 1 shows a summary of the demographics of the HVA population.

### 3.2. Absolute Signal Differences (ASD)—Raw and 10-s Decimated Data

In both the raw data and 10-s decimated data obtained using 1 Hz and 250 Hz sampling frequencies, the ASD of rSO_2_ between the NIRS systems was ~31.5% while its MAD was ~2%. In the 10-s decimated data using 1 Hz and 250 Hz sampled data, the ASD of COx-a was 0.26 while its MAD was 0.16. Overall, the ASDs of rSO_2_ were not close, which means that the INVOS NIRS system has a complicated method of deriving the rSO_2_ value, hidden under their proprietary algorithm, compared to how we derived it using the OxyMon system. Additionally, the ASD of the derived COx-a and its MAD using rSO_2_ values from both NIRS systems at both frequencies were high compared to the index’s range. Table 2 provides the results of signal disparity between the NIRS systems.

### 3.3. Pearson Correlation Analysis Results—Raw and 10-s Decimated Data

Overall, the Pearson correlation analysis between both NIRS systems showed statistically significant (median *p*-value < 0.05), very poor correlation strength (median *r* < 0.14) using raw data and 10-s decimated data at both 1 Hz and 250 Hz sampling frequencies. This analysis suggests that the measured rSO_2_ and derived COx-a signals using both NIRS systems have no linear correlation. Table 3 provides the results of Pearson correlation analysis between signals from both NIRS systems.

### 3.4. Bland–Altman Analysis Results—Raw and 10-s Decimated Data

Overall, the Bland–Altman analysis between both NIRS systems showed that the rSO_2_ and COx-a signals had a very large relative bias with a large LoA spread using both raw data and 10-s decimated data at both 1 Hz and 250 Hz. The median regression slope was near 1 for rSO_2_ and 0.05 for COx-a. This analysis suggests that the measured rSO_2_ and derived COx-a signals using both NIRS systems have poor agreement, as shown by a large relative bias, a large LoA spread, and a non-zero regression slope. Table 4 provides the results of Bland–Altman analysis between signals from both NIRS systems.

### 3.5. Cross-Correlation Function Results—Raw and 10-s Decimated Data

Overall, the cross-correlation function analysis between both NIRS systems showed that the median best lag, where the correlation value peaks, is zero using raw data and 10.5 using 10-s decimated data at both 1 Hz and 250 Hz sampling frequencies. The best lag IQR of the rSO_2_ cross-correlation was always 0, but the COx-a best lag IQR range varied. This analysis suggests that the measured rSO_2_ and derived COx-a signals using both NIRS systems are generally similar without shifting, but the derived COx-a signal has high variability. Table 5 provides the median and IQR of the best lag for the cross-correlation function analysis between signals from both NIRS systems.

### 3.6. Optimal ARIMA Structure Analysis Results—First-Order Differenced Data

Before deriving the optimal ARIMA structure, each signal’s stationarity was assessed in both frequencies. Not all physiologic signals were strictly stationary according to the ADF and KPSS tests. These signals were made strictly stationary by first-order differencing (d-order set as one) and then rechecked for their stationarity using the ADF and KPSS tests. Table 6 provides the general results for the ADF and KPSS testing on non-differenced and first-order differenced rSO_2_ and COx-a data, while File S2 shows the ADF and KPSS p-values of each signal for all subjects. Note, NA indicates that the stationarity could not be determined in the subjects due to an inadequate number of data points.

Each subject’s optimal ARIMA models for every signal were found separately for both frequencies using the lowest AIC value, and a median optimal ARIMA model was determined for each signal. It can be seen that the median optimal ARIMA model tends to stay in the lower ARIMA model range for all physiologic signals. Additionally, the median and IQR of the optimal ARIMA models of rSO_2_ and COx-a signals from the OxyMon NIRS system were simpler compared to those signals from the INVOS NIRS system. This is more pronounced when comparing the IQR range of optimal ARIMA models for the COx-a from both NIRS systems. Table 7 shows the compiled results of the median optimal and IQR of optimal ARIMA models for the combination of each signal and the sampled frequencies of data. File S3 shows a subject example of AIC recorded while fitting various ARIMA models and an example of the optimal ARIMA model for each signal per subject in both frequencies.

### 3.7. Signal Difference in Impulse Response Function (IRF) of ABP on rSO_2_—First-Order Differenced Data

The responsiveness of bivariate VARIMA IRF models was checked for variations in signal disparity between the two NIRS systems. In general, there was no significant difference in the IRF responses of ABP on rSO_2_ between the OxyMon and INVOS NIRS devices using both frequencies. It was found that the percentage of subjects showing a greater response than 0.1% was mostly above 80% of the population in either direction for the ABP and rSO_2_ signal combinations from both NIRS devices. However, a higher magnitude was often seen in the impulse response of ABP on rSO_2_ than in the alternative. There seems to be a decrease in the percentage of subjects that showed a response above 0.1% when using 1 Hz sampled data compared to 250 Hz sampled data. Table 8 provides the IRF results of signal responsiveness for signals from both NIRS devices.

### 3.8. Signal Difference in Granger Causality Relationships between ABP and rSO_2_—First-Order Differenced Data

Using 250 Hz sampled data, there were differences in the Granger causality testing results between the NIRS devices for ABP and rSO_2_ relationships. However, minor differences, less than 10%, in Granger causality testing results were seen using both 1 Hz and 250 Hz sampled data for both NIRS devices. The directional relationship of ABP leading to a directional change in rSO_2_ was often favored, but the results did not reach significance. Table 9 provides the results of the Granger causal directionality analysis for signals from both NIRS devices.

### 3.9. Subgroup Analysis Assessment

The subgroup analysis was carried out using the subgroup indicators outlined in the Methods (Section 2.5.8), with File S4 containing all the results outlined in detail. Using 1 Hz sampled data, the subgroup signal disparity analysis between the rSO_2_ and COx-a signals from the two NIRS modalities showed similar ASD, along with MAD, as seen in File S4a. This suggests that in all perturbations, the signals measured or derived using both NIRS modalities showed a higher difference compared to their range. Both the Pearson correlation and Bland–Altman agreement subgroup analyses demonstrated statistically significant nonlinear correlation between signals from both NIRS modalities, with poor agreement regardless of the perturbation type, as seen in Tables S4b and S4c, respectively. The cross-correlation function subgroup analysis suggested the rSO_2_ signal from both NIRS devices was generally similar without shift, but the COx-a signal showed high variability in all perturbation groups and had a median best lag near zero, except for the neurovascular coupling perturbation group, as seen in File S4d.

The optimal ARIMA models for every signal were found separately for each perturbation at both frequencies using the lowest AIC value. Then, a median optimal ARIMA model was determined for each combination of signal and perturbation type, as shown in File S4e. These results suggest that the median optimal ARIMA models were generally higher in the orthostatic challenge subgroup, followed by neurovascular coupling, vascular chemo-reactivity, and lowest in the baseline subgroup. Using the optimal ARIMA model subgroups, the responsiveness of bivariate VARIMA IRF models during each perturbation was checked for variations in signal disparity between the two NIRS systems. Although there was no significant difference in IRF responses for ABP on rSO_2_ or vice versa between the signals from the two modalities, the baseline group showed that ~65% of subjects showed a response greater than 0.1% in either direction of the ABP and rSO_2_ signal combination from both NIRS devices, while the response in all the perturbation groups was generally greater than the baseline, as seen in File S4f. Lastly, the Granger causality subgroup analysis showed a minor difference between the ABP and rSO_2_ relationships for both NIRS devices in all subgroups with an inconclusive directional relationship, as seen in File S4g. Similar results were found using 250 Hz sampled data.

Overall, across all categories, there were minimal differences between the results using the full recording data and subgrouped data based on baseline vs. perturbation testing of subtypes. Of note, the baseline subgroup in both frequencies tended to have the simplest median optimal ARIMA model (with p-order = 1) for all physiological signals and across all subgroups. A similar pattern of slightly lower median optimal ARIMA models was seen for signals obtained from the OxyMon NIRS system compared to the INVOS NIRS system.

## 4. Discussion

To explore and assess the difference in the signal information provided by low and high sampling frequency NIRS systems, we employed various statistical methods, including: A. Absolute differences between each modality’s rSO_2_ and COx-a signals; B. Pearson correlation, Bland–Altman agreement, and cross-correlation function comparison between each modality’s rSO_2_ and COx-a signals; C. optimal time-series autocorrelative structure differences between each modality’s rSO_2_ and COx-a signals; D. time-series impulse response function differences for ABP and rSO_2_ relationships between the NIRS systems; and E. differences in Granger causality relationships between ABP and rSO_2_ using both NIRS systems. With such an analytic approach, this is the most comprehensive analysis of signal disparity between commercial (i.e., readily available in healthcare settings) and research-grade (i.e., unavailable in healthcare settings) cerebral NIRS data to our knowledge. Through the exhaustive analysis, some important information regarding signal disparity between both NIRS systems and the effect of down-sampling and up-sampling is highlighted.

First, information on signal concordance pertaining to linear trend, magnitude proximity, linear trend, value similarity, and temporal alignment was achieved using ASD, Pearson correlation, Bland–Altman agreement, and cross-correlation function analyses, respectively. The ASD, Pearson correlation, and Bland–Altman agreement analyses using both the raw data and 10-s decimated data suggested that the rSO_2_ and COx-a signals measured/derived using either NIRS systems were not similar according to the magnitude proximity shown in the ASD analysis, with statistically significant nonlinear correlation shown in the Pearson correlation analysis, and very poor agreement shown in the Bland–Altman agreement analysis. Although temporal alignment between rSO_2_ signals from both NIRS systems was seen using a median best lag near zero in the cross-correlation function analysis, the derived COx-a signal had a non-zero best lag with high variability, which may indicate temporal misalignment between the CVR index derived from both modalities. This suggests that assuming a direct 1:1 relationship between the OxyMon and INVOS systems, or other research vs. commercial systems, is unwise. One reason for this signal disparity between both NIRS devices could be that the OxyMon NIRS signals contain more physiological information with the inclusion of pulse waveforms compared to the INVOS NIRS signals obtained at 1 Hz. Signal comparisons were performed between the NIRS devices using raw data and 10-s decimated data, with removal of pulse and respiratory frequencies, and major differences in these statistical analysis results were found. This suggests that the INVOS NIRS system is performing some type of heavy smoothing, using its proprietary algorithm, mainly focusing on lower frequency rSO_2_, as the signal concordance of correlation and agreement between the INVOS signals to the 10-s decimated OxyMon signals is low and very poor, respectively. Additionally, the different transmitter–receiver optode distances between the NIRS systems might play a role in the magnitude differences between their measured and derived signals. Similarly, if more than a simple difference of short from normal channel was performed by the INVOS NIRS proprietary algorithm to remove the extracranial signal, this may lead to critical disparities in the raw and processed data streams seen between the two NIRS platforms. The significance of these results is that these two NIRS devices cannot be used interchangeably in clinical practice since this could lead to misinterpretation of rSO_2_ and the derived COx-a signals, which require device-specific baselines and thresholds.

Second, the median optimal ARIMA models of signals from the OxyMon NIRS system were slightly simpler than the INVOS NIRS system when looking at the p-order, though they were overall comparable between the OxyMon and INVOS systems, suggesting some similar information may be carried in the rSO_2_ and COx-a metrics of the systems. These ARIMA model findings imply that both devices are likely capturing the core aspects of cerebral hemoglobin fluctuations that are most likely driven by the same vascular process, despite tangible differences in actual values reported and statistical signal structures from each respective NIRS platform. This was emphasized in the subgroup analysis across all subgroups. Additionally, the subgroup analysis indicated that all the perturbation subgroups (excluding the baseline subgroup) have p-orders ranging from 2 to 6 in the optimal ARIMA models. This indicates that perturbing the system tends to increase the autoregressive structure of the NIRS signals compared to the baseline using both NIRS systems. An interpretation of the overall observed simpler ARIMA structure using the OxyMon system compared to the INVOS system can be attributed to OxyMon’s higher sampling rate, where fewer lags were needed to capture the signal dynamics than the up-sampled INVOS signals. Additionally, a similar ARIMA structure of the OxyMon signals was seen when down-sampling to the lower frequency, which indicates that mean down-sampling seems to retain signal information at low frequencies. As with the previous work from our lab, signals from both NIRS devices were required to be first-order differenced, as shown by the non-stationarity of the non-differenced signals using ADF and KPSS stationarity tests in Table 6 [42,47,51].

Third, VARIMA IRF and Granger causality tests were conducted using both NIRS devices to compare bivariate cerebral physiologic relationships between the rSO_2_ obtained from each system and the non-invasive ABP signal and assess the lagged temporal effect of a transient rise and the directional predictability, respectively, between signals to determine their similarities or differences across both devices. The VARIMA IRF response showed an inconclusive directionality relationship between the normalized first-order differenced ABP and rSO_2_ signals from both NIRS devices by assessing whether the impulse response was greater than 0.1%. Additionally, a higher initial magnitude was often seen in the impulse responses of ABP on rSO_2_ from both systems than in the other direction. While the Granger causality analysis often favored the directional relationship of rSO_2_ Granger-caused ABP over the inverse using both systems, a very low percentage reached significance. The inconclusive directionality results of the VARIMA IRF and Granger causality analyses might be due to the use of the entire recording, with inclusion of multiple perturbations, and comparable results seen in the subgroup analysis could be due to the reduced data length included in each subgroup. However, comparing the OxyMon and INVOS systems, they appeared to perform similarly in IRF and Granger testing, with the previously mentioned cross-correlation analysis suggesting insignificant time lags between recorded physiologic information. This collectively suggests that though the raw rSO_2_ and COx-a data for research- and commercial-grade systems may be grossly different, some aspects of temporal statistical behavior are similar between the systems. As such, despite the systems not being interchangeable for the purpose of cerebral physiologic monitoring, their data streams (from raw and derived metrics) may carry some similar information at a fundamental level.

Finally, there was no significant difference between the results of the 1 Hz down-sampled data using the mean and the 250 Hz up-sampled data using the various statistical analyses employed. This suggests that mean down-sampling can retain statistical features, and simplification of high-resolution physiological signals may be needed to achieve a more manageable data size and increase computational efficiency. Hence, the rSO_2_ and COx-a signals obtained/derived from the OxyMon research-grade NIRS system are different from the same signals obtained/derived from the commercial, low sampling rate INVOS NIRS system. A reason for this could be that the high-frequency NIRS system containing full waveform data streams may contain features, even at a down-sampled 1 Hz frequency using the mean, that are not able to be captured using the low-frequency NIRS system. It offers a deeper insight into brain hemodynamics, facilitates the ability to derive more complex cerebral physiologic metrics, such as blood flow regulation, and performs time–frequency domain analysis, such as continuous wavelet transform [56], to enable the detection of subtle oscillatory patterns. These analytical approaches can deepen our understanding of interconnected relationships between cardiac cycles, respiratory rhythms, vascular slow waves, and Mayer waves for cerebral oxygen delivery and cerebral flow/flow regulation through derived CVR metrics. Furthermore, the presence of pulsatile waves in high-frequency fNIRS theoretically facilitates the reconstruction of pulse waveforms of both ICP and CBF. As such, data streams from these streams carry potential for the development of non-invasive surrogates for cerebral oxygen delivery measures, such as ICP, CBF, and CBv, with the potential to increase spatial resolution, which is not possible with invasive techniques. Current literature also supports the feasibility of extracting respiratory rate [57] and heart rate with variability [40] from high-frequency NIRS signals, along with enabling signal quality assessment and better representation of motion artifacts [57,58,59]. This analysis fosters a sense of optimism toward the ability of a high-frequency NIRS system to first correctly assess hemispheric disparity in healthy populations with perturbations, along with cranial trauma populations, and then evaluate the ability of point and interval prediction using linear ARIMA techniques. The NIRS technology has the potential to guide and advance precision medicine with growing support from the current body of literature [22,26,29,31,32,33,34,35,37,38,39,60], although further validation in other healthy populations is required at a higher sampling rate.

## 5. Limitations

As this was an exploratory study, it inherently carries several overarching limitations. First, the analysis was conducted on a reasonably sized cohort of healthy volunteers, which exceeded the minimum of 40 participants for time-domain CA studies [61]. As such, these findings should be considered exploratory and be taken with caution. Second, validation in larger high-frequency, multi-center physiological datasets is required since these findings may not be generalized to other healthy populations without perturbations. Third, we were unable to comment on the computation method of the commercial INVOS NIRS rSO_2_ signal due to the use of a proprietary algorithm in the calculation of the rSO_2_ value. Without complete transparency in the method used for extracranial tissue contamination management to derive rSO_2_, the signal disparity findings compared to the research-grade OxyMon NIRS system need to be taken with caution. Fourth, ARIMA and VARIMA are linear models and may not be able to fully capture the complexity of physiological signals, indicating the need for possibly nonlinear or hybrid approaches for more complex time-series modeling. Finally, the IRF analysis using VARIMA models and Granger causality analysis assumes linear inter-relatability of signals, while nonlinear dynamics and causal interactions remain undetected in the absence of specialized extensions.

## 6. Conclusions

The rSO_2_ and COx-a signals obtained from the high-frequency OxyMon NIRS system contain different and possibly better signal features compared to the same signals obtained/derived from the low-frequency INVOS NIRS system. The various statistical methods employed (ASD, Pearson correlation, Bland–Altman agreement, cross-correlation function, optimal time-series autocorrelative structure, time-series impulse response function, and Granger causality relationships) pointed toward this conclusion. A possibility of the signal disparity found between the two NIRS modalities in this analysis could be that the signal features contained in the higher sampling rate NIRS are not being fully captured in the lower sampling rate NIRS signals. As such, the newer high-frequency NIRS systems, offering full waveform data, seem to have a brighter future for the derivation of non-invasive cerebral CVR indices that can be assessed in various healthy populations, along with cranial trauma populations.

## Figures and Tables

**Table 1:** Demographic data for HVA population.

Variable	Median [IQR] or Count (%)
Duration of Recording (min)	89.5 (85.7–95.3)
Number of Subjects	50
Age (years)	28 (23.3–35)
Biological Sex (Male)	28 (56%)
Hand Dominance (Right)	49 (98%)

The table depicts the summary of demographic data for the HVA population. HVA, healthy volunteer.

**Table 2:** Signal disparity analysis between NIRS OxyMon and INVOS system signals.

Physiologic Variable	Median (IQR)	
	Raw Data	10-s Decimated Data
1 Hz Sampled Data		
ASD of rSO_2_ (%)	31.85 (28.52–34.34)	31.56 (28.65–34.43)
ASD of COx-a (au)	–	0.26 (0.12–0.47)
MAD of ASD rSO_2_ (%)	2.2 (1.88–2.73)	2.16 (1.75–2.7)
MAD of ASD COx-a (au)	–	0.16 (0.13–0.19)
250 Hz Sampled Data		
ASD of rSO_2_ (%)	31.36 (28.08–34.64)	31.48 (28.71–34.39)
ASD of COx-a (au)	–	0.26 (0.12–0.46)
MAD of ASD rSO_2_ (%)	2.26 (1.94–3.01)	2.18 (1.74–2.67)
MAD of ASD COx-a (au)	–	0.16 (0.14–0.19)

The table shows the absolute signal disparity analysis between two NIRS system signals (OxyMon and INVOS) using raw and 10-s decimated data at 1 Hz and 250 Hz sampling frequencies. ASD, absolute signal difference; au, arbitrary units; COx-a, cerebral oximetry index with arterial blood pressure; IQR, interquartile range; MAD, median absolute deviation; rSO_2_, regional cerebral oxygen saturation.

**Table 3:** Pearson correlation analysis between NIRS OxyMon and INVOS system signals.

Physiologic Variable	Value	Median (IQR)	
		Raw Data	10-s Decimated Data
1 Hz Sampled Data			
rSO_2_	*r*	0.14 (−0.03–0.27)	0.14 (−0.05–0.33)
	*p*	4.0 × 10^−50^ (3.2 × 10^−231^–2.5 × 10^−10^)	1.6 × 10^−9^ (1.0 × 10^−16^–0.01)
COx-a	*r*	–	0.08 (−0.07–0.19)
	*p*	–	1.20 × 10^−3^ (4.8 × 10^−9^–0.07)
250 Hz Sampled Data			
rSO_2_	*r*	0.07 (−0.02–0.26)	0.14 (−0.05–0.33)
	*p*	0 (0–0)	2.5 × 10^−7^ (9.1 × 10^−16^–0.01)
COx-a	*r*	–	0.08 (−0.07–0.18)
	*p*	–	3.0 × 10^−3^ (2.9 × 10^−9^–0.08)

The table shows the Pearson correlation analysis between two NIRS system signals (OxyMon and INVOS) using raw and 10-s decimated data at 1 Hz and 250 Hz sampling frequencies. COx-a, cerebral oximetry index with arterial blood pressure; IQR, interquartile range; MAD, median absolute deviation; *r*-value, Pearson correlation coefficient; rSO_2_, regional cerebral oxygen saturation.

**Table 4:** Bland–Altman analysis between NIRS OxyMon and INVOS system signals.

Physiologic Variable	Value	Median (IQR)	
		Raw Data	10-s Decimated Data
1 Hz Sampled Data			
rSO_2_	Bias	31.57 (21.31–35.79)	31.53 (21.45–35.24)
	Lower LoA	23.89 (12.1–27.93)	24.53 (12.68–28.66)
	Upper LoA	38.96 (27.48–43.9)	38.87 (27.26–43.14)
	LoA Spread	12.47 (11.47–16.43)	12.33 (10.96–15.82)
	Relative Bias	208.05 (154.14–287.69)	230.51 (161.51–310.5)
	Regression Slope	0.96 (0.66–1.57)	1.1 (0.72–1.63)
	Regression Intercept	−29.56 (−58.07–−3.86)	−31.08 (−63.86–−8.67)
COx-a	Bias	–	−0.05 (−0.16–0.01)
	Lower LoA	–	−0.78 (−1.03–−0.7)
	Upper LoA	–	0.74 (0.65–0.84)
	LoA Spread	–	1.55 (1.4–1.8)
	Relative Bias	–	−3.03 (−10.04–0.85)
	Regression Slope	–	0.05 (−0.17–0.18)
	Regression Intercept	–	−0.04 (−0.14–0.02)
250 Hz Sampled Data			
rSO_2_	Bias	31.49 (21.31–35.73)	31.51 (21.44–35.17)
	Lower LoA	22.11 (10.04–27.39)	23.69 (12.68–28.06)
	Upper LoA	39.1 (28.61–44.42)	38.86 (27.25–43.64)
	LoA Spread	13.2 (11.69–21.09)	12.32 (10.91–15.92)
	Relative Bias	191.27 (107.95–278.3)	211.72 (154.9–310.2)
	Regression Slope	0.86 (−0.33–1.49)	1.06 (0.69–1.59)
	Regression Intercept	−21.73 (−53.7–54.47)	−29.38 (−63.67–−4.91)
COx-a	Bias	–	−0.05 (−0.17–0.02)
	Lower LoA	–	−0.78 (−1.03–−0.7)
	Upper LoA	–	0.74 (0.65–0.85)
	LoA Spread	–	1.57 (1.4–1.8)
	Relative Bias	–	−3.37 (−9.62–1.08)
	Regression Slope	–	0.05 (−0.15–0.21)
	Regression Intercept	–	−0.04 (−0.16–0.02)

The table shows the Bland–Altman analysis between two NIRS system signals (OxyMon and INVOS) using raw and 10-s decimated data at 1 Hz and 250 Hz sampling frequencies. COx-a, cerebral oximetry index with arterial blood pressure; IQR, interquartile range; LoA, limit of agreement; rSO_2_, regional cerebral oxygen saturation.

**Table 5:** Cross-correlation function best lag between NIRS OxyMon and INVOS system signals.

Physiologic Variable	Best Lag [Median (IQR)]	
	Raw Data	10-s Decimated Data
1 Hz Sampled Data		
rSO_2_	0 (0–0)	0 (0–0)
COx-a	–	10.5 (−76.5–87)
250 Hz Sampled Data		
rSO_2_	0 (0–0)	0 (0–0)
COx-a	–	10.5 (−83–105)

The table shows the cross-correlation function best lag between two NIRS system signals (OxyMon and INVOS) using raw and 10-s decimated data at 1 Hz and 250 Hz sampling frequencies. COx-a, cerebral oximetry index with arterial blood pressure; IQR, interquartile range; rSO_2_, regional cerebral oxygen saturation.

**Table 6:** ADF and KPSS results showing stationarity vs non-stationarity vs NA for physiologic signals.

**ADF results for non-differenced data**															
**Frequency**	**ABP**			**rSO_2__Invos**			**COx-a_Invos**			**rSO_2__OxyMon**			**COx-a_OxyMon**		
	**S**	**NS**	**NA**	**S**	**NS**	**NA**	**S**	**NS**	**NA**	**S**	**NS**	**NA**	**S**	**NS**	**NA**
1 Hz	49	1	0	36	14	0	48	2	0	38	12	0	50	0	0
250 Hz	49	1	0	37	13	0	48	2	0	39	11	0	48	2	0
**ADF results for first-order differenced data**															
**Frequency**	**ABP**			**rSO_2__Invos**			**COx-a_Invos**			**rSO_2__OxyMon**			**COx-a_OxyMon**		
	**S**	**NS**	**NA**	**S**	**NS**	**S**	**NS**	**NA**	**S**	**NS**	**S**	**NS**	**NA**	**S**	**NS**
1 Hz	50	0	0	50	0	0	50	0	0	50	0	0	50	0	0
250 Hz	50	0	0	50	0	0	50	0	0	50	0	0	50	0	0
**KPSS results for non-differenced data**															
**Frequency**	**ABP**			**rSO_2__Invos**			**COx-a_Invos**			**rSO_2__OxyMon**			**COx-a_OxyMon**		
	**S**	**NS**	**NA**	**S**	**NS**		**S**	**NS**	**NA**	**S**	**NS**	**NA**	**S**	**NS**	**NA**
1 Hz	22	28	0	9	41	0	31	19	0	8	42	0	40	10	0
250 Hz	21	29	0	9	41	0	31	19	0	8	42	0	40	10	0
**KPSS results for first-order differenced data**															
**Frequency**	**ABP**			**rSO_2__Invos**			**COx-a_Invos**			**rSO_2__OxyMon**			**COx-a_OxyMon**		
	**S**	**NS**	**NA**	**S**	**NS**		**S**	**NS**	**NA**	**S**	**NS**	**NA**	**S**	**NS**	**NA**
1 Hz	49	1	0	50	0	0	50	0	0	50	0	0	50	0	0
250 Hz	48	2	0	50	0	0	50	0	0	50	0	0	50	0	0

The table presents the results of the ADF and KPSS analysis with the count of subject data that were found to be stationary (S), non-stationary (NS), or not possible to assess (NA) using non-differenced and first-order differenced data for all populations. It was found from these stationarity tests that signals were stationary after first-order differencing, while they were originally non-stationary. ADF, augmented Dickey–Fuller; COx, cerebral oximetry index with cerebral perfusion pressure; COx-a, cerebral oximetry index with arterial blood pressure; HC, healthy control volunteer group; KPSS, Kwiatkowski–Phillips–Schmidt–Shin; NA, unable to assess stationarity; NS, non-stationary; rSO_2_, regional cerebral oxygen saturation; S, stationary.

**Table 7:** Median and IQR of optimal ARIMA models based on AIC.

Physiologic Variable	Optimal ARIMA Models (Median [IQR])	
	1 Hz	250 Hz
ABP	(5, 1, 3) [(3, 1, 3)–(6, 1, 7)]	(5, 1, 1) [(3, 1, 3)–(7, 1, 3)]
rSO_2__Invos	(4, 1, 7) [(3, 1, 3)–(6, 1, 7)]	(4, 1, 4) [(2, 1, 2)–(6, 1, 7)]
COx-a_Invos	(3, 1, 6) [(1, 1, 10)–(6, 1, 5)]	(3, 1, 0) [(1, 1, 8)–(6, 1, 3)]
rSO_2__OxyMon	(2, 1, 3) [(1, 1, 10)–(7, 1, 1)]	(3, 1, 3) [(2, 1, 1)–(5, 1, 1)]
COx-a_ OxyMon	(2, 1, 9) [(2, 1, 0)–(4, 1, 8)]	(2, 1, 9) [(2, 1, 1)–(4, 1, 2)]

The table provides the median and IQR of optimal ARIMA models based on AIC for all physiologic variables using data at 1 Hz and 250 Hz frequencies. ABP, arterial blood pressure; AIC, Akaike information criterion; ARIMA, autoregressive integrative moving average; COx-a, cerebral oximetry index with ABP; IQR, interquartile range; rSO_2_, regional cerebral oxygen saturation.

**Table 8:** Signal responsiveness using impulse response coefficients of the optimal VARIMA model.

Direction	1 Hz [% (Count)]		250 Hz [% (Count)]	
	>0.1%	NA	>0.1%	NA
ABP → rSO_2__Invos	84% (42)	4% (2)	94% (47)	2% (1)
rSO_2__Invos → ABP	80% (40)	4% (2)	94% (47)	2% (1)
ABP → rSO_2__OxyMon	90% (45)	2% (1)	96% (48)	2% (1)
rSO_2__OxyMon → ABP	90% (45)	2% (1)	98% (49)	2% (1)

The table shows the responsiveness of signals using the impulse response coefficients of the optimal VARIMA model from INVOS and OxyMon NIRS devices. ABP, arterial blood pressure; p-order, autoregressive order; rSO_2_, regional cerebral oxygen saturation; VARIMA, vector autoregressive integrative moving average.

**Table 9:** Granger causal directionality results based on greater F-statistics.

Signal	Direction	1 Hz [% (Count)]	250 Hz [% (Count)]
ABP and rSO_2__Invos	ABP → rSO_2__Invos	54% (27)	52% (26)
	rSO_2__Invos → ABP	46% (23)	48% (24)
ABP and rSO_2__OxyMon	ABP → rSO_2__OxyMon	50% (25)	52% (26)
	rSO_2__OxyMon → ABP	50% (25)	48% (24)

The table shows the directional relationship between ABP and rSO_2_ using the INVOS and OxyMon NIRS devices. ABP, arterial blood pressure; p-order, autoregressive order; rSO_2_, regional cerebral oxygen saturation.

## Data Availability

Research ethics board approval at our institution does not facilitate the free and open sharing of human data, regardless of the data being in a de-identified fashion. All such data are protected under both ethics and privacy acts within the Province of Manitoba, preventing such open sharing of data. All the data analyzed and used are available from the corresponding author upon reasonable request.

## Supplementary Materials

The following supporting information can be downloaded at: https://www.mdpi.com/article/10.3390/s25175391/s1, File S1: Methodology Appendix [42,47,51,52,53,54]; File S2: Time-Series Stationarity Analysis; File S3: Autoregressive Integrative Moving Average (ARIMA) Analysis; File S4: Subgroup Analyses.

## Abbreviations

The following abbreviations are used in this manuscript:

α	Alpha
ABP	Arterial blood pressure
ADF	Augmented Dickey–Fuller
AIC	Akaike information criterion
ARIMA	Autoregressive integrative moving average
ASD	Absolute signal difference
CA	Cerebral autoregulation
CBF	Cerebral blood flow
CBv	Cerebral blood volume
COx	Cerebral oximetry index with CPP
COx-a	Cerebral oximetry index with ABP
COx-a_Invos	COx-a obtained with rSO_2_ from the INVOS NIRS system
COx-a_Oxymon	COx-a obtained with rSO_2_ from the OxyMon NIRS system
CPP	Cerebral perfusion pressure
CVR	Cerebrovascular reactivity
d-order	Moving average order
fNIRS	Functional near-infrared spectroscopy
HbDiff	Difference between oxyhemoglobin and deoxyhemoglobin
HbO	Oxyhemoglobin
HHb	Deoxyhemoglobin
HVA	Healthy volunteers
ICP	Intracranial pressure
IQR	Interquartile range
IRF	Impulse response function
KPSS	Kwiatkowski–Phillips–Schmidt–Shin
LoA	Limit of agreement
MAIN-HUB	Multi-Omic Analytics and Integrative Neuroinformatics in the HUman Brain
MAD	Median absolute deviation
NA	Represents missing value or undetermined value due to inadequate data
NIRS	Near-infrared spectroscopy
p-order	Autoregressive order
q-order	Moving average order
*r*	Pearson correlation coefficient
relative bias	Bias as a proportion of LoA spread
rSO_2_	Regional cerebral oxygen saturation
rSO_2__Invos	Frontal right rSO_2_ signal obtained using the INVOS NIRS system
rSO_2__Oxymon	Frontal right rSO_2_ signal obtained using the OxyMon NIRS system
tHb	Total hemoglobin
VARIMA	Vector ARIMA
