# Supplementary material for: Time-Domain Analysis of Low- and High-Frequency Near-Infrared Spectroscopy Sensor Technologies for Characterization of Cerebral Pressure–Flow and Oxygen Delivery Physiology: A Prospective Observational Study
Source: Sensors (Basel). 2025 Sep 1;25(17):5391. doi: 10.3390/s25175391 (PMC12430896; doi:10.3390/s25175391)
Supplement: Supplementary file 1 [file sensors-25-05391-s001.zip › File S1.pdf]

File S1 – Methodology Appendix

File S1 – Table of Contents

File S1a: Median and IQR of Relative HHb and tHb Signals from Normal and Short Channels using 1Hz Sampled Data 1

File S1b: Evaluation of Data Stationarity 4

File S1c: Evaluation of optimal Autoregressive Integrated Moving Average (ARIMA) orders 4

File S1d: Generation of Vector Autoregressive Integrated Moving Average (VARIMA) model 5

File S1e: References 5

File S1a: Median and IQR of Relative HHb and tHb Signals from Normal and Short Channels using 1Hz Sampled Data

Subject/ Median	Normal Ch HHb	Normal Ch tHb	Short Ch HHb	Short Ch tHb
1	74.1 (64.18 – 76.96)	176.88 (162.35 – 184.06)	149.25 (140.68 – 152.97)	368.29 (347.4 – 379.18)
2	81.38 (80.44 – 82.75)	192.16 (189.89 – 197.18)	156.46 (149.1 – 164.07)	384.29 (371.97 – 400.05)
3	61.46 (59.97 – 62.29)	139.24 (136.59 – 141)	116.72 (114.69 – 119.13)	300.74 (294.78 – 307.53)
4	80.06 (79.22 – 80.65)	181.71 (179.87 – 182.73)	202.12 (193.75 – 213.62)	441.65 (422.37 – 461.01)
5	61.86 (59.9 – 62.79)	154.23 (152.94 – 156.12)	121.76 (116.76 – 122.82)	308.29 (299.92 – 311.62)
6	61.57 (60.99 – 62.32)	144.3 (141.24 – 150.12)	142.91 (136.47 – 144.33)	334.44 (327.01 – 348.24)

7	73.6 (72.8 – 74.97)	188.47 (186.14 – 189.99)	149.48 (147.69 – 155.24)	373.26 (370.04 – 393.31)
8	97.55 (96.66 – 98.51)	238.66 (237.28 – 241)	244.44 (242.33 – 246.04)	553.88 (550.65 – 558.62)
9	95.83 (95.19 – 97.61)	210.48 (208.56 – 215.89)	267.92 (259.57 – 292.62)	582.58 (569.69 – 611.88)
10	88.96 (85.99 – 91.43)	207.76 (204.09 – 217.28)	133.56 (129.88 – 135.78)	353.04 (345.68 – 360.34)
11	34.83 (34.83 – 34.83)	97.44 (97.44 – 97.44)	111.86 (104.98 – 112.79)	295.69 (291.59 – 299.27)
12	122.74 (115.25 – 124.12)	256.45 (252.31 – 267.68)	378.87 (355.51 – 389.86)	767.56 (747.56 – 780.12)
13	52.97 (50.31 – 54.54)	127.81 (124.83 – 131.83)	116.95 (114.16 – 118.37)	301.17 (295.8 – 312.92)
14	138.51 (136.18 – 140.82)	315.65 (313.34 – 318.2)	285.45 (280.8 – 300.48)	587.91 (572.56 – 607.43)
15	34.83 (34.83 – 34.83)	97.44 (97.44 – 97.44)	123.15 (118.99 – 139.72)	329.46 (312.87 – 347.99)
16	50.91 (49.96 – 53.1)	115.85 (112.95 – 120.47)	153.94 (144.56 – 157.72)	354.77 (337.18 – 362.53)
17	56.97 (56.4 – 57.58)	136.87 (135.18 – 138.74)	110.53 (104.36 – 118.88)	293.73 (291.4 – 308.26)
18	98.75 (96.29 – 100.64)	230.44 (228.18 – 234.17)	252.15 (242.97 – 280.99)	587.03 (580.33 – 613.01)
19	119.82 (113.73 – 125)	267.49 (259.96 – 275.81)	327.7 (316.23 – 354.04)	726.06 (689.61 – 745.31)
20	34.83 (34.83 – 34.83)	97.44 (97.44 – 97.44)	103.87 (103.86 – 108.05)	291.24 (291.24 – 305.18)
21	43.28 (42.35 – 44.2)	113 (111.77 – 114.18)	103.87 (103.86 – 104.42)	291.24 (291.24 – 291.41)
22	71.51 (69.98 – 74.41)	173.78 (171.08 – 177.69)	196.24 (194.37 – 197.38)	478.43 (472.53 – 483.11)
23	45.44 (43.54 – 46.91)	108.86 (104.28 – 114.54)	117.13 (113.76 – 121.53)	299.31 (294.39 – 307.04)
24	80.55 (75.08 – 84.83)	198.25 (196 – 203.3)	256.6 (209.34 – 382.29)	584.33 (494.12 – 1034.9)
25	61.01 (60.59 – 61.47)	163.22 (161.56 – 165.06)	132.54 (129.66 – 136.93)	347.37 (337.85 – 355.27)
26	43.89 (43.1 – 44.71)	117.39 (116.35 – 119.91)	112.22 (106.98 – 115.57)	295 (292.22 – 304.32)

27	65.45 (55.94 – 67.67)	163.32 (141.38 – 167.45)	108.67 (106.72 – 114.04)	297.63 (292.66 – 306.81)
28	68.9 (66.99 – 76.72)	163.48 (160.41 – 183.2)	109.13 (106.35 – 121.33)	292.9 (292.03 – 310.67)
29	69.22 (68.49 – 70.61)	172.97 (171.87 – 175.32)	157.95 (150.73 – 159.69)	388.07 (369.07 – 395.84)
30	88.69 (58.18 – 92.95)	208.3 (149.2 – 218.45)	214.69 (104.66 – 243.95)	503.45 (302.51 – 548.68)
31	62.9 (61.26 – 64.7)	147.96 (145.24 – 150.57)	123.8 (121.24 – 145.62)	298.64 (296.8 – 345.8)
32	127.18 (125.26 – 129.08)	343.8 (342.15 – 345.49)	381.57 (375.54 – 387.45)	1033.22 (1028.45 – 1038.11)
33	40.66 (39.98 – 41.53)	114.01 (112.31 – 116.12)	107 (105.99 – 108.26)	298.05 (295.18 – 303.08)
34	64.41 (62.55 – 66.52)	154.46 (152.09 – 161.08)	133.17 (127.81 – 139.06)	339.63 (333.49 – 356.89)
35	54.26 (53.41 – 56.35)	135.38 (133.49 – 140.56)	104.06 (103.86 – 109.17)	291.3 (291.24 – 293.09)
36	69.17 (65.44 – 72.46)	157.12 (145.39 – 166.22)	189.37 (185.75 – 191.86)	452.94 (446.66 – 459.51)
37	98.97 (97.16 – 100.09)	235.85 (233.2 – 245.71)	300.63 (268.92 – 334.56)	682.6 (612.83 – 744.47)
38	56.28 (55.79 – 56.91)	122.11 (121.15 – 123.57)	142.02 (139.27 – 149.43)	339.01 (333.88 – 353)
39	81 (79.36 – 84.85)	187.14 (180.88 – 193.59)	227.35 (220.97 – 239.97)	532.55 (512.88 – 543.31)
40	109.57 (106.37 – 131.58)	256.21 (246 – 320.94)	222.38 (215.13 – 258.26)	523.18 (514.76 – 605.52)
41	94.55 (91.97 – 96.58)	214.99 (204.24 – 218.86)	227 (222.16 – 233.2)	514.03 (505.52 – 525.5)
42	105.33 (100.03 – 107.46)	236.44 (231.64 – 240.06)	343.07 (290.14 – 369.85)	768.75 (661.07 – 816.91)
43	93.73 (84.91 – 103.68)	216.54 (195.02 – 245.11)	240.26 (234.16 – 250.72)	577.15 (565.79 – 588.26)
44	113.64 (110 – 115.99)	264.1 (248.92 – 268.65)	265.51 (257.36 – 281.55)	597.33 (584.4 – 627.08)
45	99.96 (96.81 – 102.57)	233.48 (231.32 – 238.72)	252.23 (245.68 – 259.28)	580.15 (576.32 – 588.86)
46	45.66 (44.67 – 46.06)	113.7 (111.69 – 115.21)	125.02 (123.23 – 140.61)	307.6 (303.73 – 346.26)

47	42.96 (42.24 – 43.36)	103.16 (101.37 – 104.98)	119.79 (117.98 – 125.85)	300.64 (296.07 – 314.03)
48	93.07 (91.93 – 97.8)	202.51 (201.16 – 218.01)	220.13 (213.75 – 229.32)	509 (493.18 – 520.89)
49	103.07 (100.66 – 106.02)	244.65 (236.98 – 254.9)	256.17 (246.17 – 272)	574.31 (548.17 – 624.22)
50	51.74 (43.84 – 52.8)	119.1 (102.01 – 123.91)	127.52 (121.53 – 128.97)	323.37 (315.9 – 329.41)
Median	70.36 (66.22 – 1.69)	173.38 (161.95 – 180.21)	151.71 (142.62 – 156.48)	370.78 (346.54 – 386.24)

File S1b: Evaluation of Data Stationarity

Stationarity analysis was performed for each physiologic signal at an individual level of each population using Augmented Dickey-Fuller (ADF) and Kwiatkowski-Phillips-Schmidt-Shin (KPSS) tests in 1Hz and 250Hz sampling frequencies, in keeping with previous work from our group. The ADF test informs if the series is trend-stationary and KPSS test informs if the series is stationary around a linear trend [1]. The ADF and KPSS tests were run on each patient data using the "adfuller" and "kpss" functions from the *statsmodels* package (https://www.statsmodels.org/stable/index.html) with an example of patient results shown for each dataset given in File S2a-c. The data from populations were 1st order differenced, and the above-mentioned stationarity analysis was re-run on differenced data in both sampling frequencies, with an example of patient results for each dataset shown in File S2a-c.

File S1c: Evaluation of optimal Autoregressive Integrated Moving Average (ARIMA) orders

Using Python, Autoregressive Integrated Moving Average (ARIMA) model fit was performed in accordance with standard Box-Jenkin's time-series methodologies [1–3]. Various ARIMA models were fit to each univariate physiologic time-series to model every 1st order differenced signal for each patient in 1Hz and 250Hz sampling frequencies. The Akaike Information Criterion (AIC) values were obtained for ARIMA model fit on all right frontal regional oxygen saturation (rSO_2) and cerebral oximetry index derived with arterial blood pressure (COx-a) obtained using OxyMon and INVOS near-infrared spectroscopy systems

since it is neither stringent or lenient as compared to other performance values [4–6]. Based on previous research from our lab [4–6], the list of ARIMA models were obtained by varying the autoregressive order (p-order), and the moving average order (q-order) from 1 to 10, and 0 to 10, respectively. The integrative order (d-order) was separately varied from 0 to 1 using the previously described differencing method. An example of these outputs is shown in File S3a. To find the optimal ARIMA model for each physiologic time-series in various temporal resolutions, the lowest AIC values were extracted for a patient. Finally, by reordering the optimal ARIMA models sequentially, the median optimal ARIMA model for a physiologic signal in each temporal resolution was found using AIC and an example for the population is given in File S3b.

File S1d: Generation of Vector Autoregressive Integrated Moving Average (VARIMA) model

To represent the relationship between two types of signals, we derived vector ARIMA (VARIMA) models. The VARIMA p-order was calculated by taking a product of the previously saved optimal ARIMA p-orders for the two signals being evaluated and the VARIMA q-order was calculated by adding the previously saved optimal ARIMA q-orders for the two signals being evaluated, as suggested from past literature [3]. The VARIMA d-order was equal to one since 1st order differenced data was used. This created a personalized VARIMA model for each 1st order differenced signal pair in a subject's data for 1Hz and 250Hz sampling frequencies.

File S1e: References

1. Chatfield, C.; Xing, H. *The Analysis of Time Series: An Introduction with R*; 7th ed.; Chapman and Hall/CRC, 2019; ISBN 978-1-138-06613-7.
2. Chatfield, C. *The Analysis of Time Series: An Introduction*; 6th ed.; Chapman and Hall/CRC: New York, 2003; ISBN 978-0-429-20870-6.

3. Lütkepohl, H. *New Introduction to Multiple Time Series Analysis*; New York : Springer: Berlin, 2005; ISBN 978-3-540-40172-8.
4. Sainbhi, A.S.; Vakitbilir, N.; Gomez, A.; Stein, K.Y.; Froese, L.; Zeiler, F.A. Time-Series Autocorrelative Structure of Cerebrovascular Reactivity Metrics in Severe Neural Injury: An Evaluation of the Impact of Data Resolution. *Biomedical Signal Processing and Control* **2024**, *95*, 106403, doi:10.1016/j.bspc.2024.106403.
5. Sainbhi, A.S.; Froese, L.; Stein, K.Y.; Vakitbilir, N.; Gomez, A.; Islam, A.; Bergmann, T.; Silvaggio, N.; Hayat, M.; Zeiler, F.A. Commercial NIRS May Not Detect Hemispheric Regional Disparity in Continuously Measured COx/COx-a: An Exploratory Healthy and Cranial Trauma Time-Series Analysis. *Bioengineering* **2025**, *12*, 247, doi:10.3390/bioengineering12030247.
6. Sainbhi, A.S.; Froese, L.; Stein, K.Y.; Vakitbilir, N.; Hasan, R.; Gomez, A.; Bergmann, T.; Silvaggio, N.; Hayat, M.; Moon, J.; et al. Time-Series Autoregressive Models for Point and Interval Forecasting of Raw and Derived Commercial Near-Infrared Spectroscopy Measures: An Exploratory Cranial Trauma and Healthy Control Analysis. *Bioengineering* **2025**, doi:10.3390/bioengineering12070682.
