# Supplementary material for: Time-Domain Analysis of Low- and High-Frequency Near-Infrared Spectroscopy Sensor Technologies for Characterization of Cerebral Pressure–Flow and Oxygen Delivery Physiology: A Prospective Observational Study
Source: Sensors (Basel). 2025 Sep 1;25(17):5391. doi: 10.3390/s25175391 (PMC12430896; doi:10.3390/s25175391)
Supplement: Supplementary file 1 [file sensors-25-05391-s001.zip › File S2.pdf]

File S2 – Cross-Correlation Function Analysis

File S2 – Table of Contents

File S2a: ADF and KPSS P-Values for Non-Differenced and 1st Order Differenced Data in 1Hz Sampling Frequency 2

File S2b: ADF and KPSS P-Values for Non-Differenced and 1st Order Differenced Data in 250Hz Sampling Frequency 4

File S2a: ADF and KPSS P-Values for Non-Differenced and 1st Order Differenced Data in 1Hz Sampling Frequency

**ADF p-values for 1Hz Sampled Data**										
**Subject**	***Non-Differenced***					***1st Order Differenced***				
	**ABP**	**rSO_2_Invos**	**COx-a_Invos**	**rSO_2_Oxymon**	**COx-a_OxyMon**	**ABP**	**rSO_2_Invos**	**COx-a_Invos**	**rSO_2_Oxymon**	**COx-a_OxyMon**
1	0.00	0.00	0.00	0.00	0.00	0.00	0.00	0.00	0.00	0.00
2	0.00	0.04	0.00	0.02	0.01	0.00	0.00	0.00	0.00	0.00
3	0.00	0.00	0.02	0.00	0.00	0.00	0.00	0.00	0.00	0.00
4	0.00	0.05	0.03	0.00	0.00	0.00	0.00	0.00	0.00	0.00
5	0.00	0.01	0.04	0.00	0.00	0.00	0.00	0.00	0.00	0.00
6	0.00	0.00	0.00	0.05	0.01	0.00	0.00	0.00	0.00	0.00
7	0.00	0.15	0.00	0.19	0.01	0.00	0.00	0.00	0.00	0.00
8	0.00	0.09	0.01	0.00	0.00	0.00	0.00	0.00	0.00	0.00
9	0.00	0.32	0.02	0.10	0.01	0.00	0.00	0.00	0.00	0.00
10	0.00	0.00	0.00	0.00	0.00	0.00	0.00	0.00	0.00	0.00
11	0.00	0.14	0.00	0.03	0.01	0.00	0.00	0.00	0.00	0.00
12	0.00	0.07	0.01	0.15	0.00	0.00	0.00	0.00	0.00	0.00
13	0.00	0.00	0.00	0.00	0.04	0.00	0.00	0.00	0.00	0.00
14	0.00	0.00	0.05	0.00	0.02	0.00	0.00	0.00	0.00	0.00
15	0.00	0.04	0.02	0.26	0.00	0.00	0.00	0.00	0.00	0.00
16	0.00	0.08	0.00	0.00	0.00	0.00	0.00	0.00	0.00	0.00
17	0.00	0.00	0.01	0.47	0.01	0.00	0.00	0.00	0.00	0.00
18	0.00	0.00	0.00	0.25	0.02	0.00	0.00	0.00	0.00	0.00
19	0.00	0.02	0.00	0.07	0.00	0.00	0.00	0.00	0.00	0.00
20	0.00	0.00	0.16	0.00	0.00	0.00	0.00	0.00	0.00	0.00
21	0.00	0.64	0.00	0.00	0.00	0.00	0.00	0.00	0.00	0.00
22	0.00	0.00	0.00	0.00	0.00	0.00	0.00	0.00	0.00	0.00
23	0.00	0.00	0.00	0.01	0.00	0.00	0.00	0.00	0.00	0.00
24	0.00	0.00	0.01	0.73	0.03	0.00	0.00	0.00	0.00	0.00
25	0.00	0.22	0.00	0.00	0.00	0.00	0.00	0.00	0.00	0.00
26	0.00	0.09	0.00	0.14	0.00	0.00	0.00	0.00	0.00	0.00
27	0.00	0.00	0.00	0.00	0.00	0.00	0.00	0.00	0.00	0.00
28	0.00	0.01	0.00	0.31	0.05	0.00	0.00	0.00	0.00	0.00
29	0.00	0.00	0.00	0.00	0.00	0.00	0.00	0.00	0.00	0.00
30	0.00	0.00	0.00	0.25	0.00	0.00	0.00	0.00	0.00	0.00
31	0.00	0.01	0.00	0.00	0.00	0.00	0.00	0.00	0.00	0.00
32	0.00	0.11	0.00	0.00	0.01	0.00	0.00	0.00	0.00	0.00
33	0.00	0.11	0.01	0.01	0.00	0.00	0.00	0.00	0.00	0.00
34	0.21	0.00	0.01	0.04	0.00	0.00	0.00	0.00	0.00	0.00
35	0.00	0.01	0.00	0.00	0.00	0.00	0.00	0.00	0.00	0.00
36	0.00	0.00	0.00	0.00	0.00	0.00	0.00	0.00	0.00	0.00
37	0.00	0.08	0.00	0.00	0.01	0.00	0.00	0.00	0.00	0.00
38	0.00	0.05	0.00	0.01	0.00	0.00	0.00	0.00	0.00	0.00
39	0.00	0.00	0.01	0.01	0.00	0.00	0.00	0.00	0.00	0.00
40	0.00	0.01	0.00	0.00	0.02	0.00	0.00	0.00	0.00	0.00
41	0.00	0.00	0.00	0.00	0.00	0.00	0.00	0.00	0.00	0.00
42	0.00	0.01	0.00	0.00	0.00	0.00	0.00	0.00	0.00	0.00
43	0.00	0.00	0.00	0.00	0.00	0.00	0.00	0.00	0.00	0.00
44	0.00	0.01	0.06	0.00	0.01	0.00	0.00	0.00	0.00	0.00
45	0.00	0.00	0.00	0.06	0.00	0.00	0.00	0.00	0.00	0.00
46	0.00	0.01	0.00	0.00	0.01	0.00	0.00	0.00	0.00	0.00
47	0.00	0.01	0.00	0.00	0.00	0.00	0.00	0.00	0.00	0.00
48	0.00	0.00	0.02	0.00	0.00	0.00	0.00	0.00	0.00	0.00
49	0.00	0.14	0.01	0.03	0.00	0.00	0.00	0.00	0.00	0.00
50	0.00	0.01	0.01	0.00	0.00	0.00	0.00	0.00	0.00	0.00
**KPSS p-values for 1Hz Sampled Data**										

Subject	Non-Differenced					1st Order Differenced				
	ABP	rSO_2_Invos	COx-a_Invos	rSO_2_Oxymon	COx-a_OxyMon	ABP	rSO_2_Invos	COx-a_Invos	rSO_2_Oxymon	COx-a_OxyMon
1	0.01	0.10	0.04	0.01	0.05	0.10	0.10	0.10	0.10	0.10
2	0.01	0.01	0.07	0.01	0.10	0.10	0.10	0.10	0.10	0.10
3	0.10	0.01	0.05	0.01	0.01	0.08	0.10	0.10	0.10	0.10
4	0.02	0.01	0.05	0.10	0.10	0.10	0.10	0.10	0.10	0.10
5	0.01	0.01	0.10	0.01	0.10	0.10	0.10	0.10	0.10	0.10
6	0.06	0.01	0.10	0.01	0.10	0.10	0.10	0.10	0.10	0.10
7	0.01	0.01	0.10	0.01	0.05	0.10	0.10	0.10	0.10	0.10
8	0.04	0.01	0.10	0.10	0.10	0.10	0.10	0.10	0.10	0.10
9	0.10	0.01	0.10	0.01	0.10	0.10	0.10	0.10	0.10	0.10
10	0.10	0.06	0.04	0.01	0.10	0.10	0.10	0.10	0.10	0.10
11	0.01	0.01	0.10	0.01	0.10	0.10	0.10	0.10	0.10	0.10
12	0.01	0.01	0.07	0.01	0.10	0.10	0.10	0.10	0.10	0.10
13	0.10	0.01	0.10	0.01	0.05	0.10	0.10	0.10	0.10	0.10
14	0.01	0.01	0.02	0.01	0.10	0.10	0.10	0.10	0.10	0.10
15	0.01	0.01	0.10	0.01	0.04	0.10	0.10	0.10	0.10	0.10
16	0.01	0.01	0.01	0.10	0.10	0.10	0.10	0.10	0.10	0.10
17	0.01	0.01	0.02	0.01	0.02	0.10	0.10	0.10	0.10	0.10
18	0.10	0.01	0.10	0.01	0.10	0.10	0.10	0.10	0.10	0.10
19	0.10	0.01	0.10	0.01	0.10	0.10	0.10	0.10	0.10	0.10
20	0.10	0.08	0.01	0.03	0.10	0.10	0.10	0.10	0.10	0.10
21	0.10	0.01	0.02	0.01	0.10	0.10	0.10	0.10	0.10	0.10
22	0.04	0.10	0.10	0.08	0.10	0.10	0.10	0.10	0.10	0.10
23	0.04	0.01	0.10	0.01	0.10	0.10	0.10	0.10	0.10	0.10
24	0.10	0.09	0.03	0.01	0.05	0.10	0.10	0.10	0.10	0.10
25	0.10	0.01	0.10	0.01	0.10	0.10	0.10	0.10	0.10	0.10
26	0.01	0.01	0.10	0.01	0.10	0.10	0.10	0.10	0.10	0.10
27	0.10	0.01	0.10	0.01	0.10	0.10	0.10	0.10	0.10	0.10
28	0.01	0.01	0.10	0.01	0.10	0.10	0.10	0.10	0.10	0.10
29	0.01	0.01	0.10	0.10	0.10	0.10	0.10	0.10	0.10	0.10
30	0.01	0.01	0.10	0.01	0.10	0.10	0.10	0.10	0.10	0.10
31	0.06	0.01	0.01	0.01	0.10	0.10	0.10	0.10	0.10	0.10
32	0.01	0.01	0.10	0.10	0.10	0.10	0.10	0.10	0.10	0.10
33	0.01	0.01	0.04	0.01	0.02	0.10	0.10	0.10	0.10	0.10
34	0.01	0.10	0.01	0.10	0.10	0.10	0.10	0.10	0.10	0.10
35	0.10	0.01	0.10	0.01	0.10	0.10	0.10	0.10	0.10	0.10
36	0.01	0.01	0.10	0.01	0.10	0.10	0.10	0.10	0.10	0.10
37	0.10	0.01	0.10	0.03	0.10	0.10	0.10	0.10	0.10	0.10
38	0.01	0.01	0.10	0.01	0.01	0.10	0.10	0.10	0.10	0.10
39	0.10	0.09	0.01	0.01	0.10	0.10	0.10	0.10	0.10	0.10
40	0.10	0.10	0.10	0.01	0.10	0.10	0.10	0.10	0.10	0.10
41	0.01	0.01	0.02	0.07	0.08	0.10	0.10	0.10	0.10	0.10
42	0.03	0.01	0.03	0.04	0.10	0.10	0.10	0.10	0.10	0.10
43	0.10	0.01	0.10	0.02	0.10	0.10	0.10	0.10	0.10	0.10
44	0.01	0.01	0.10	0.01	0.10	0.10	0.10	0.10	0.10	0.10
45	0.01	0.01	0.02	0.01	0.01	0.10	0.10	0.10	0.10	0.10
46	0.01	0.06	0.01	0.01	0.10	0.04	0.10	0.10	0.10	0.10
47	0.10	0.01	0.10	0.01	0.10	0.10	0.10	0.10	0.10	0.10
48	0.08	0.04	0.01	0.01	0.10	0.10	0.10	0.10	0.10	0.10
49	0.10	0.01	0.06	0.01	0.10	0.10	0.10	0.10	0.10	0.10
50	0.10	0.01	0.01	0.01	0.01	0.10	0.10	0.10	0.10	0.10

ABP, arterial blood pressure; ADF, Augmented Dickey-Fuller; COx-a, cerebral oximetry index with ABP; KPSS, Kwiatkowski-Phillips-Schmidt-Shin; rSO_2, regional cerebral oxygen saturation.

File S2b: ADF and KPSS P-Values for Non-Differenced and 1st Order Differenced Data in 250Hz Sampling Frequency

ADF p-values for 250Hz Sampled Data										
Subject	*Non-Differenced*					*1st Order Differenced*				
	ABP	rSO_2_Invos	COx-a_Invos	rSO_2_Oxymon	COx-a_OxyMon	ABP	rSO_2_Invos	COx-a_Invos	rSO_2_Oxymon	COx-a_OxyMon
1	0.00	0.00	0.00	0.00	0.00	0.00	0.00	0.00	0.00	0.00
2	0.00	0.03	0.00	0.02	0.01	0.00	0.00	0.00	0.00	0.00
3	0.00	0.00	0.02	0.00	0.00	0.00	0.00	0.00	0.00	0.00
4	0.00	0.05	0.03	0.00	0.01	0.00	0.00	0.00	0.00	0.00
5	0.00	0.01	0.04	0.00	0.00	0.00	0.00	0.00	0.00	0.00
6	0.00	0.00	0.00	0.03	0.01	0.00	0.00	0.00	0.00	0.00
7	0.00	0.14	0.00	0.18	0.01	0.00	0.00	0.00	0.00	0.00
8	0.00	0.13	0.01	0.00	0.00	0.00	0.00	0.00	0.00	0.00
9	0.00	0.30	0.02	0.11	0.01	0.00	0.00	0.00	0.00	0.00
10	0.00	0.00	0.00	0.00	0.00	0.00	0.00	0.00	0.00	0.00
11	0.00	0.14	0.00	0.03	0.01	0.00	0.00	0.00	0.00	0.00
12	0.00	0.04	0.01	0.21	0.00	0.00	0.00	0.00	0.00	0.00
13	0.00	0.00	0.00	0.00	0.05	0.00	0.00	0.00	0.00	0.00
14	0.00	0.00	0.05	0.00	0.05	0.00	0.00	0.00	0.00	0.00
15	0.00	0.04	0.02	0.30	0.00	0.00	0.00	0.00	0.00	0.00
16	0.00	0.07	0.00	0.00	0.00	0.00	0.00	0.00	0.00	0.00
17	0.00	0.00	0.01	0.47	0.01	0.00	0.00	0.00	0.00	0.00
18	0.00	0.00	0.00	0.21	0.02	0.00	0.00	0.00	0.00	0.00
19	0.00	0.02	0.00	0.07	0.00	0.00	0.00	0.00	0.00	0.00
20	0.00	0.00	0.17	0.00	0.00	0.00	0.00	0.00	0.00	0.00
21	0.00	0.65	0.00	0.00	0.00	0.00	0.00	0.00	0.00	0.00
22	0.00	0.00	0.00	0.00	0.00	0.00	0.00	0.00	0.00	0.00
23	0.00	0.00	0.00	0.00	0.00	0.00	0.00	0.00	0.00	0.00
24	0.00	0.00	0.01	0.73	0.05	0.00	0.00	0.00	0.00	0.00
25	0.00	0.25	0.00	0.00	0.00	0.00	0.00	0.00	0.00	0.00
26	0.00	0.06	0.01	0.16	0.00	0.00	0.00	0.00	0.00	0.00
27	0.00	0.00	0.00	0.00	0.01	0.00	0.00	0.00	0.00	0.00
28	0.00	0.00	0.00	0.27	0.03	0.00	0.00	0.00	0.00	0.00
29	0.00	0.00	0.00	0.00	0.00	0.00	0.00	0.00	0.00	0.00
30	0.00	0.00	0.00	0.02	0.00	0.00	0.00	0.00	0.00	0.00
31	0.00	0.01	0.00	0.00	0.00	0.00	0.00	0.00	0.00	0.00
32	0.00	0.12	0.01	0.00	0.00	0.00	0.00	0.00	0.00	0.00
33	0.00	0.10	0.01	0.01	0.01	0.00	0.00	0.00	0.00	0.00
34	0.23	0.00	0.01	0.04	0.00	0.00	0.00	0.00	0.00	0.00
35	0.00	0.01	0.00	0.00	0.00	0.00	0.00	0.00	0.00	0.00
36	0.00	0.00	0.00	0.00	0.00	0.00	0.00	0.00	0.00	0.00
37	0.00	0.07	0.00	0.00	0.01	0.00	0.00	0.00	0.00	0.00
38	0.00	0.04	0.00	0.00	0.00	0.00	0.00	0.00	0.00	0.00
39	0.00	0.00	0.01	0.01	0.00	0.00	0.00	0.00	0.00	0.00
40	0.00	0.01	0.00	0.00	0.01	0.00	0.00	0.00	0.00	0.00
41	0.00	0.02	0.00	0.00	0.00	0.00	0.00	0.00	0.00	0.00
42	0.00	0.01	0.01	0.00	0.00	0.00	0.00	0.00	0.00	0.00
43	0.00	0.00	0.02	0.00	0.00	0.00	0.00	0.00	0.00	0.00
44	0.00	0.01	0.06	0.00	0.02	0.00	0.00	0.00	0.00	0.00
45	0.00	0.00	0.00	0.06	0.00	0.00	0.00	0.00	0.00	0.00
46	0.00	0.01	0.00	0.00	0.01	0.00	0.00	0.00	0.00	0.00
47	0.00	0.01	0.00	0.00	0.00	0.00	0.00	0.00	0.00	0.00
48	0.00	0.00	0.02	0.00	0.00	0.00	0.00	0.00	0.00	0.00
49	0.00	0.14	0.01	0.03	0.00	0.00	0.00	0.00	0.00	0.00
50	0.00	0.01	0.02	0.00	0.00	0.00	0.00	0.00	0.00	0.00
**KPSS p-values for 250Hz Sampled Data**										

Subject	*Non-Differenced*					*1st Order Differenced*				
	**ABP**	**rSO_2_Invos**	**COx-a_Invos**	**rSO_2_Oxymon**	**COx-a_OxyMon**	**ABP**	**rSO_2_Invos**	**COx-a_Invos**	**rSO_2_Oxymon**	**COx-a_OxyMon**
1	0.01	0.10	0.04	0.01	0.04	0.10	0.10	0.10	0.10	0.10
2	0.01	0.01	0.08	0.01	0.10	0.10	0.10	0.10	0.10	0.10
3	0.10	0.01	0.07	0.01	0.01	0.10	0.10	0.10	0.10	0.10
4	0.02	0.01	0.06	0.10	0.10	0.10	0.10	0.10	0.10	0.10
5	0.01	0.01	0.10	0.01	0.10	0.10	0.10	0.10	0.10	0.10
6	0.06	0.01	0.10	0.01	0.10	0.10	0.10	0.10	0.10	0.10
7	0.01	0.01	0.10	0.01	0.05	0.10	0.10	0.10	0.10	0.10
8	0.04	0.01	0.10	0.10	0.10	0.10	0.10	0.10	0.10	0.10
9	0.10	0.01	0.10	0.01	0.10	0.10	0.10	0.10	0.10	0.10
10	0.10	0.06	0.03	0.01	0.10	0.10	0.10	0.10	0.10	0.10
11	0.01	0.01	0.10	0.01	0.10	0.10	0.10	0.10	0.10	0.10
12	0.01	0.01	0.08	0.01	0.10	0.10	0.10	0.10	0.10	0.10
13	0.10	0.01	0.10	0.01	0.06	0.10	0.10	0.10	0.10	0.10
14	0.01	0.01	0.02	0.01	0.10	0.10	0.10	0.10	0.10	0.10
15	0.01	0.01	0.10	0.01	0.03	0.10	0.10	0.10	0.10	0.10
16	0.01	0.01	0.01	0.10	0.10	0.10	0.10	0.10	0.10	0.10
17	0.01	0.01	0.03	0.01	0.02	0.10	0.10	0.10	0.10	0.10
18	0.10	0.01	0.10	0.01	0.10	0.10	0.10	0.10	0.10	0.10
19	0.10	0.01	0.09	0.01	0.10	0.10	0.10	0.10	0.10	0.10
20	0.10	0.08	0.01	0.03	0.10	0.10	0.10	0.10	0.10	0.10
21	0.10	0.01	0.02	0.01	0.10	0.10	0.10	0.10	0.10	0.10
22	0.03	0.10	0.10	0.08	0.10	0.10	0.10	0.10	0.10	0.10
23	0.04	0.01	0.10	0.01	0.10	0.10	0.10	0.10	0.10	0.10
24	0.10	0.10	0.02	0.01	0.04	0.10	0.10	0.10	0.10	0.10
25	0.10	0.01	0.10	0.01	0.10	0.10	0.10	0.10	0.10	0.10
26	0.01	0.01	0.10	0.01	0.10	0.10	0.10	0.10	0.10	0.10
27	0.10	0.01	0.10	0.01	0.10	0.10	0.10	0.10	0.10	0.10
28	0.01	0.01	0.08	0.01	0.10	0.10	0.10	0.10	0.10	0.10
29	0.01	0.01	0.09	0.10	0.10	0.10	0.10	0.10	0.10	0.10
30	0.01	0.01	0.10	0.01	0.10	0.10	0.10	0.10	0.10	0.10
31	0.05	0.01	0.01	0.01	0.10	0.10	0.10	0.10	0.10	0.10
32	0.01	0.01	0.10	0.10	0.10	0.10	0.10	0.10	0.10	0.10
33	0.01	0.01	0.04	0.01	0.02	0.10	0.10	0.10	0.10	0.10
34	0.01	0.10	0.01	0.10	0.10	0.10	0.10	0.10	0.10	0.10
35	0.10	0.01	0.10	0.02	0.10	0.10	0.10	0.10	0.10	0.10
36	0.01	0.01	0.10	0.01	0.10	0.04	0.10	0.10	0.10	0.10
37	0.10	0.01	0.10	0.03	0.10	0.10	0.10	0.10	0.10	0.10
38	0.01	0.01	0.10	0.01	0.01	0.10	0.10	0.10	0.10	0.10
39	0.10	0.09	0.02	0.01	0.10	0.10	0.10	0.10	0.10	0.10
40	0.10	0.10	0.10	0.01	0.10	0.10	0.10	0.10	0.10	0.10
41	0.01	0.01	0.01	0.06	0.08	0.10	0.10	0.10	0.10	0.10
42	0.03	0.01	0.04	0.05	0.10	0.10	0.10	0.10	0.10	0.10
43	0.10	0.01	0.10	0.02	0.10	0.10	0.10	0.10	0.10	0.10
44	0.01	0.01	0.10	0.01	0.10	0.10	0.10	0.10	0.10	0.10
45	0.01	0.01	0.02	0.01	0.01	0.10	0.10	0.10	0.10	0.10
46	0.01	0.06	0.01	0.01	0.10	0.04	0.10	0.10	0.10	0.10
47	0.10	0.01	0.10	0.01	0.10	0.10	0.10	0.10	0.10	0.10
48	0.08	0.04	0.01	0.01	0.10	0.10	0.10	0.10	0.10	0.10
49	0.10	0.01	0.05	0.01	0.10	0.10	0.10	0.10	0.10	0.10
50	0.10	0.01	0.02	0.01	0.01	0.10	0.10	0.10	0.10	0.10

ABP, arterial blood pressure; ADF, Augmented Dickey-Fuller; COx-a, cerebral oximetry index with ABP; KPSS, Kwiatkowski-Phillips-Schmidt-Shin; rSO_2, regional cerebral oxygen saturation.
