# Supplementary material for: Time-Domain Analysis of Low- and High-Frequency Near-Infrared Spectroscopy Sensor Technologies for Characterization of Cerebral Pressure–Flow and Oxygen Delivery Physiology: A Prospective Observational Study
Source: Sensors (Basel). 2025 Sep 1;25(17):5391. doi: 10.3390/s25175391 (PMC12430896; doi:10.3390/s25175391)
Supplement: Supplementary file 1 [file sensors-25-05391-s001.zip › File S3.pdf]

File S3 – Autoregressive Integrative Moving Average (ARIMA) Analysis

File S3 – Table of Contents

File S3a: Recorded AIC while fitting various ARIMA models – Subject Example using 250Hz Sampled Data 2

File S3b: Optimal ARIMA Models based on AIC – 250Hz Sampled Data Example 4

File S3a: Recorded AIC while fitting various ARIMA models – Subject Example using 250Hz Sampled Data

ARIMA Model	ABP	rSO_2_Invos	COx-a_Invos	rSO_2_OxyMon	COx-a_OxyMon
(1,1,0)	4039.7913	1270.8145	-1374.7050	94.8068	-1461.0870
(1,1,1)	3896.3854	1266.3124	-1373.0302	95.4500	-1459.5824
(1,1,2)	3892.9354	1252.2712	-1371.1324	72.7075	-1457.6104
(1,1,3)	3890.3897	1253.9641	-1374.6308	74.7159	-1460.1512
(1,1,4)	3893.2982	1252.9190	-1369.2757	76.9776	-1455.4096
(1,1,5)	3895.2454	1254.6804	-1367.1166	73.3343	-1453.3608
(1,1,6)	3886.7655	1256.2455	-1365.2705	75.3342	-1453.6153
(1,1,7)	3889.1931	1258.1794	-1372.9131	77.3148	-1452.3952
(1,1,8)	3897.8710	1266.8387	-1376.8267	79.1785	-1451.4995
(1,1,9)	3890.6178	1260.6470	-1382.7690	80.6301	-1453.2101
(1,1,10)	3894.8214	1262.5835	-1378.6230	84.1260	-1451.9801
(2,1,0)	3979.6089	1261.6835	-1373.0619	93.8225	-1459.4564
(2,1,1)	3894.1879	1260.9498	-1371.0714	72.9989	-1462.0957
(2,1,2)	3892.4725	1263.0059	-1369.0927	91.4743	-1460.1025
(2,1,3)	3893.3153	1256.2521	-1369.4969	76.3709	-1458.0950
(2,1,4)	3885.1455	1257.6251	-1367.2732	78.3974	-1454.7773
(2,1,5)	3891.5439	1256.4018	-1365.5648	75.7095	-1452.3588
(2,1,6)	3887.7333	1266.3534	-1363.6046	77.2263	-1455.5932
(2,1,7)	3890.4807	1260.1480	-1378.5884	79.0409	-1455.5416
(2,1,8)	3898.5053	1268.0922	-1376.2007	80.9681	-1454.5927
(2,1,9)	3891.0704	1270.0875	-1364.2038	82.9966	-1451.2667
(2,1,10)	3898.0505	1271.8555	-1379.2036	85.5862	-1462.2261
(3,1,0)	3953.8293	1261.0481	-1371.0936	95.2910	-1457.6647
(3,1,1)	3894.1923	1262.9654	-1369.0710	95.9381	-1460.1157
(3,1,2)	3893.4541	1259.7590	-1367.4173	77.1043	-1458.3597
(3,1,3)	3895.3134	1257.6980	-1372.3240	74.5662	-1456.4010
(3,1,4)	3893.2523	1254.2987	-1365.7428	74.8322	-1452.2454
(3,1,5)	3898.3233	1256.3044	-1376.9835	77.3451	-1458.5425
(3,1,6)	3892.3658	1255.9089	-1361.5506	79.2562	-1453.5410
(3,1,7)	3894.5442	1258.6813	-1375.0975	79.3771	-1453.9596
(3,1,8)	3901.9149	1256.8685	-1378.9511	81.2773	-1452.7193
(3,1,9)	3900.7586	1258.2962	-1356.6201	83.1435	-1457.9680
(3,1,10)	3899.9956	1264.8244	-1376.6440	84.0116	-1460.2099
(4,1,0)	3929.3005	1262.9817	-1369.4348	97.0303	-1457.2741
(4,1,1)	3893.6309	1264.9648	-1370.0010	97.4768	-1455.2804
(4,1,2)	3893.6253	1256.0907	-1366.9782	78.9379	-1466.2657
(4,1,3)	3890.1726	1261.9424	-1364.9760	75.1737	-1454.2578
(4,1,4)	3894.9685	1256.5199	-1363.5280	75.2850	-1453.2490
(4,1,5)	3898.7823	1258.3997	-1375.7731	77.3902	-1450.8954
(4,1,6)	3895.5514	1261.5043	-1360.1246	81.2546	-1465.6327
(4,1,7)	3889.8826	1261.5285	-1359.0383	81.5981	-1455.5009
(4,1,8)	3893.2110	1263.0226	-1357.2411	83.0498	-1457.6394
(4,1,9)	3893.5702	1263.1270	-1353.3128	85.9190	-1458.1458
(4,1,10)	3890.2089	1269.8792	-1375.2699	87.0242	-1460.4937
(5,1,0)	3925.3534	1264.7811	-1369.1925	91.8653	-1455.2743
(5,1,1)	3888.5140	1254.4256	-1367.6088	75.0629	-1453.2826
(5,1,2)	3891.4357	1256.4687	-1365.5097	77.3893	-1459.0771
(5,1,3)	3887.0235	1258.6347	-1363.7034	75.2602	-1452.7611
(5,1,4)	3898.6768	1262.9675	-1362.2341	77.3923	-1451.2591
(5,1,5)	3895.2207	1260.6267	-1375.9792	83.7449	-1448.0212
(5,1,6)	3892.0385	1261.2071	-1358.0919	83.9553	-1460.5428
(5,1,7)	3889.6697	1259.9466	-1356.8028	82.2199	-1458.9560
(5,1,8)	3908.3996	1264.2348	-1355.3221	86.6013	-1456.6541
(5,1,9)	3896.7531	1266.3954	-1352.0909	86.8008	-1456.9257
(5,1,10)	3896.0789	1268.1060	-1376.7175	86.6130	-1460.3734
(6,1,0)	3915.8698	1264.1419	-1367.3076	92.3582	-1453.9467

(6,1,1)	3890.2657	1256.4076	-1365.6149	75.9181	-1452.6685
(6,1,2)	3888.5402	1257.4292	-1363.7051	79.0624	-1451.8223
(6,1,3)	3897.6199	1258.6790	-1361.7047	79.0219	-1449.2141
(6,1,4)	3897.7368	1260.9252	-1361.0526	80.7419	-1451.4161
(6,1,5)	3888.4192	1262.8484	-1358.4399	84.7269	-1445.0918
(6,1,6)	3892.7163	1265.6228	-1356.1329	83.3267	-1449.7048
(6,1,7)	3896.4752	1265.1712	-1355.0925	81.5880	-1463.9350
(6,1,8)	3893.7252	1267.7799	-1354.7139	85.1667	-1460.8746
(6,1,9)	3894.7941	1258.7105	-1350.7093	86.5995	-1445.2866
(6,1,10)	3894.7347	1258.3853	-1375.1307	85.1431	-1457.6534
(7,1,0)	3914.5169	1264.6696	-1366.2681	92.6510	-1453.9114
(7,1,1)	3889.1654	1266.3201	-1364.2693	77.8983	-1451.9116
(7,1,2)	3890.7394	1260.0442	-1363.4828	79.5163	-1453.0889
(7,1,3)	3895.7481	1269.9008	-1360.9691	80.2609	-1451.0425
(7,1,4)	3895.0548	1270.6062	-1358.7132	81.3510	-1448.6923
(7,1,5)	3890.2684	1264.6686	-1356.6308	83.6853	-1446.7739
(7,1,6)	3895.7205	1267.0316	-1355.2903	79.9514	-1446.5809
(7,1,7)	3894.4666	1267.2047	-1352.8521	84.7131	-1463.0320
(7,1,8)	3896.1778	1265.0957	-1352.5236	86.9125	-1460.2560
(7,1,9)	3894.4385	1261.9907	-1348.2026	88.8961	-1444.1559
(7,1,10)	3892.4365	1265.1012	-1373.9083	87.3223	-1459.0166
(8,1,0)	3913.2069	1265.8511	-1364.2721	88.0861	-1451.9120
(8,1,1)	3890.9647	1267.5756	-1362.2699	79.8984	-1449.9450
(8,1,2)	3889.7353	1260.0666	-1361.0250	81.0471	-1453.7544
(8,1,3)	3894.2281	1265.7888	-1358.9822	81.3482	-1452.0109
(8,1,4)	3895.9281	1269.3971	-1356.7078	83.6450	-1448.0705
(8,1,5)	3894.0441	1265.4186	-1354.6576	85.5500	-1447.3127
(8,1,6)	3895.6698	1265.5941	-1353.6060	80.8786	-1444.4744
(8,1,7)	3894.8740	1269.2543	-1355.0118	83.9468	-1459.6992
(8,1,8)	3894.7621	1270.9643	-1348.2122	89.0924	-1461.0736
(8,1,9)	3899.3689	1267.0330	-1348.1599	90.9560	-1446.1994
(8,1,10)	3895.1416	1272.1346	-1373.9355	88.1282	-1457.2314
(9,1,0)	3914.1387	1267.2195	-1363.2924	89.9022	-1453.4998
(9,1,1)	3892.6400	1269.1994	-1379.6170	80.6435	-1451.5154
(9,1,2)	3892.4442	1263.3701	-1377.8809	83.3343	-1451.9835
(9,1,3)	3894.6115	1261.8818	-1371.2869	83.3764	-1451.6628
(9,1,4)	3896.0984	1271.4947	-1358.7308	85.2074	-1448.6359
(9,1,5)	3895.4740	1264.5576	-1364.7328	87.3982	-1446.7113
(9,1,6)	3897.3999	1267.8590	-1354.7393	87.3950	-1448.5777
(9,1,7)	3897.6004	1268.8030	-1353.4031	84.5305	-1462.3943
(9,1,8)	3901.2251	1273.0506	-1350.6548	93.0162	-1443.0777
(9,1,9)	3902.7080	1267.7445	-1374.8326	93.7369	-1442.8219
(9,1,10)	3894.7788	1272.7924	-1371.8417	92.2668	-1450.9264
(10,1,0)	3914.8143	1269.1525	-1361.8034	91.7971	-1451.5437
(10,1,1)	3894.6259	1270.9184	-1361.0276	81.4163	-1449.5003
(10,1,2)	3893.3358	1263.7939	-1379.5680	82.8900	-1450.4398
(10,1,3)	3896.6673	1264.3912	-1378.4368	83.7920	-1450.1764
(10,1,4)	3897.7796	1259.2997	-1377.4388	87.5249	-1450.9940
(10,1,5)	3898.5632	1264.8936	-1371.7508	88.2034	-1449.3731
(10,1,6)	3896.1084	1270.5914	-1373.7152	89.5981	-1449.6295
(10,1,7)	3900.1884	1269.7044	-1375.1842	91.1018	-1445.6870
(10,1,8)	3901.3473	1273.1672	-1363.7220	93.1634	-1443.4430
(10,1,9)	3904.2350	1266.9821	-1377.8606	91.8502	-1441.9076
(10,1,10)	3896.5217	1278.2995	-1371.0621	96.3125	-1451.3100

ABP, arterial blood pressure; AIC, Akaike Information Criterion; ARIMA, autoregressive integrative moving average; COx-a, cerebral oximetry index with ABP; rSO_2, regional cerebral oxygen saturation.

File S3b: Optimal ARIMA Models based on AIC – 250Hz Sampled Data Example

Subject	ABP	rSO_2_Invos	COx-a_Invos	rSO_2_OxyMon	COx-a_OxyMon
1	(1,1,8)	(2,1,2)	(2,1,9)	(10,1,10)	(1,1,9)
2	(7,1,3)	(4,1,5)	(6,1,1)	(1,1,1)	(9,1,3)
3	(1,1,4)	(2,1,3)	(7,1,6)	(10,1,7)	(7,1,1)
4	(3,1,4)	(7,1,9)	(1,1,0)	(2,1,1)	(3,1,6)
5	(10,1,5)	(9,1,9)	(3,1,0)	(6,1,3)	(3,1,3)
6	(7,1,8)	(5,1,5)	(5,1,9)	(1,1,7)	(2,1,9)
7	(6,1,3)	(4,1,10)	(6,1,3)	(2,1,4)	(3,1,7)
8	(6,1,2)	(2,1,4)	(2,1,7)	(2,1,3)	(1,1,3)
9	(5,1,10)	(5,1,2)	(2,1,8)	(3,1,2)	(2,1,0)
10	(4,1,4)	(3,1,10)	(1,1,8)	(1,1,2)	(2,1,1)
11	(7,1,5)	(6,1,7)	(1,1,8)	(3,1,6)	(6,1,7)
12	(9,1,9)	(2,1,1)	(7,1,8)	(2,1,3)	(2,1,1)
13	(2,1,5)	(1,1,1)	(1,1,7)	(3,1,3)	(2,1,2)
14	(7,1,4)	(4,1,4)	(1,1,1)	(1,1,6)	(2,1,6)
15	(5,1,4)	(6,1,2)	(1,1,1)	(2,1,0)	(5,1,6)
16	(6,1,5)	(1,1,2)	(3,1,2)	(3,1,5)	(2,1,7)
17	(7,1,1)	(6,1,7)	(4,1,9)	(4,1,1)	(4,1,5)
18	(5,1,1)	(1,1,2)	(7,1,3)	(7,1,2)	(3,1,0)
19	(1,1,2)	(3,1,3)	(1,1,5)	(3,1,3)	(1,1,2)
20	(8,1,6)	(2,1,8)	(10,1,10)	(3,1,2)	(2,1,9)
21	(3,1,9)	(10,1,9)	(3,1,2)	(9,1,10)	(3,1,3)
22	(5,1,3)	(1,1,3)	(2,1,2)	(3,1,5)	(3,1,2)
23	(4,1,3)	(10,1,9)	(7,1,1)	(4,1,1)	(1,1,2)
24	(6,1,1)	(3,1,2)	(1,1,0)	(7,1,8)	(3,1,2)
25	(4,1,3)	(6,1,4)	(5,1,7)	(5,1,6)	(5,1,1)
26	(5,1,1)	(1,1,9)	(1,1,8)	(3,1,5)	(10,1,6)
27	(3,1,8)	(1,1,2)	(1,1,5)	(3,1,6)	(2,1,8)
28	(7,1,3)	(1,1,9)	(7,1,2)	(6,1,1)	(1,1,1)
29	(1,1,6)	(10,1,1)	(1,1,3)	(1,1,2)	(3,1,1)
30	(4,1,10)	(1,1,5)	(10,1,9)	(1,1,2)	(2,1,4)
31	(4,1,1)	(10,1,1)	(1,1,8)	(5,1,1)	(1,1,1)
32	(8,1,8)	(2,1,5)	(2,1,7)	(2,1,3)	(3,1,3)
33	(3,1,3)	(9,1,2)	(4,1,1)	(2,1,3)	(1,1,7)
34	(9,1,7)	(8,1,6)	(1,1,5)	(1,1,0)	(3,1,8)
35	(1,1,6)	(6,1,1)	(3,1,4)	(9,1,1)	(2,1,1)
36	(6,1,4)	(2,1,4)	(4,1,4)	(2,1,2)	(2,1,7)
37	(2,1,10)	(1,1,7)	(1,1,6)	(2,1,5)	(1,1,4)
38	(1,1,3)	(4,1,8)	(6,1,4)	(3,1,6)	(5,1,4)
39	(3,1,3)	(6,1,6)	(4,1,3)	(2,1,2)	(1,1,0)
40	(5,1,6)	(7,1,3)	(4,1,3)	(6,1,9)	(3,1,1)
41	(3,1,5)	(8,1,3)	(2,1,5)	(1,1,1)	(2,1,1)
42	(9,1,3)	(1,1,9)	(1,1,6)	(7,1,5)	(1,1,4)
43	(5,1,2)	(1,1,3)	(6,1,7)	(3,1,3)	(1,1,4)
44	(3,1,2)	(4,1,9)	(3,1,0)	(4,1,6)	(4,1,6)
45	(6,1,3)	(5,1,3)	(2,1,10)	(3,1,3)	(2,1,8)
46	(2,1,7)	(3,1,2)	(3,1,7)	(1,1,3)	(1,1,6)
47	(2,1,4)	(1,1,2)	(1,1,9)	(1,1,2)	(4,1,2)
48	(3,1,5)	(4,1,4)	(1,1,6)	(1,1,2)	(7,1,1)
49	(9,1,5)	(5,1,7)	(7,1,2)	(3,1,4)	(2,1,7)
50	(1,1,3)	(4,1,3)	(10,1,10)	(1,1,7)	(5,1,2)

ABP, arterial blood pressure; AIC, Akaike Information Criterion; ARIMA, autoregressive integrative moving average; COx-a, cerebral oximetry index with ABP; rSO_2, regional cerebral oxygen saturation.
