# Supplementary material for: Time-Domain Analysis of Low- and High-Frequency Near-Infrared Spectroscopy Sensor Technologies for Characterization of Cerebral Pressure–Flow and Oxygen Delivery Physiology: A Prospective Observational Study
Source: Sensors (Basel). 2025 Sep 1;25(17):5391. doi: 10.3390/s25175391 (PMC12430896; doi:10.3390/s25175391)
Supplement: Supplementary file 1 [file sensors-25-05391-s001.zip › File S4.pdf]

File S4 – Subgrouped Analysis

File S4 – Table of Contents

File S4a: Subgrouped Signal Disparity Analysis Between NIRS OxyMon and INVOS System Signals 2

File S4b: Subgrouped Pearson Correlation Analysis Between NIRS OxyMon and INVOS System Signals 3

File S4c: Subgrouped Bland-Altman Analysis Between NIRS OxyMon and INVOS System Signals 4

File S4d: Subgrouped Cross-Correlation Function Best Lag Between NIRS OxyMon and INVOS System Signals 5

File S4e: Subgrouped Median and IQR of Optimal ARIMA Models Based on AIC 6

File S4f: Subgrouped Signal Responsiveness using Impulse Response Coefficients of Optimal VARIMA model 7

File S4g: Subgrouped Granger Causal Directionality Results Based on Greater F-Statistic 8

File S4a: Subgrouped Signal Disparity Analysis Between NIRS OxyMon and INVOS System Signals

Physiologic Variable	Median (IQR)							
	Baseline		Neurovascular Coupling		Orthostatic Challenge		Vascular Chemo-Reactivity	
	Raw Data	10-Second Decimated Data	Raw Data	10-Second Decimated Data	Raw Data	10-Second Decimated Data	Raw Data	10-Second Decimated Data
**1Hz Sampled Data**								
**ASD of rSO_2 (%)**	32.12 (28.93 – 34.51)	32.1 (28.92 – 34.43)	33.34 (31.86 – 34.71)	33.2 (31.76 – 34.67)	31.35 (28.05 – 33.27)	31.34 (28.18 – 33.16)	27.58 (26.35 – 28.89)	27.7 (26.64 – 28.85)
**ASD of COx-a (au)**	–	0.27 (0.13 – 0.49)	–	0.27 (0.14 – 0.44)	–	0.27 (0.12 – 0.48)	–	0.19 (0.09 – 0.31)
**MAD of ASD rSO_2 (%)**	1.8 (1.39 – 2.5)	1.6 (1.19 – 2.48)	1.22 (1.01 – 1.65)	1.11 (0.85 – 1.56)	2.01 (1.72 – 2.38)	1.82 (1.55 – 2.15)	1.21 (0.96 – 1.58)	1.04 (0.76 – 1.47)
**MAD of ASD COx-a (au)**	–	0.12 (0.08 – 0.19)	–	0.15 (0.11 – 0.19)	–	0.16 (0.14 – 0.2)	–	0.09 (0.07 – 0.13)
**250Hz Sampled Data**								
**ASD of rSO_2 (%)**	32.11 (28.18 – 34.56)	32.16 (28.92 – 34.84)	33.32 (30.29 – 35.42)	33.25 (31.76 – 34.69)	30.98 (27.84 – 33.32)	31.17 (28.23 – 33.08)	27.75 (26.39 – 29.54)	27.73 (26.62 – 28.9)
**ASD of COx-a (au)**	–	0.27 (0.12 – 0.49)	–	0.28 (0.14 – 0.45)	–	0.27 (0.13 – 0.48)	–	0.19 (0.1 – 0.33)
**MAD of ASD rSO_2 (%)**	1.81 (1.36 – 2.87)	1.58 (1.2 – 2.4)	1.3 (1.04 – 1.77)	1.09 (0.86 – 1.59)	2.08 (1.76 – 2.51)	1.81 (1.53 – 2.19)	1.33 (0.94 – 1.76)	1.11 (0.8 – 1.4)
**MAD of ASD COx-a (au)**	–	0.13 (0.08 – 0.18)	–	0.14 (0.11 – 0.19)	–	0.17 (0.14 – 0.21)	–	0.1 (0.08 – 0.13)

ASD, absolute signal difference; au, arbitrary units; COx-a, cerebral oximetry index with arterial blood pressure; IQR, interquartile range; MAD, median absolute deviation; rSO_2, regional cerebral oxygen saturation.

File S4b: Subgrouped Pearson Correlation Analysis Between NIRS OxyMon and INVOS System Signals

Physiologic Variable	Value	Median (IQR)							
		Baseline		Neurovascular Coupling		Orthostatic Challenge		Vascular Chemo-Reactivity	
		Raw Data	10-Second Decimated Data	Raw Data	10-Second Decimated Data	Raw Data	10-Second Decimated Data	Raw Data	10-Second Decimated Data
**1Hz Sampled Data**									
**rSO_2**	*r*	0.16 (-0.29 – 0.4)	0.18 (-0.31 – 0.45)	-0.04 (-0.18 – 0.08)	-0.06 (-0.2 – 0.09)	2.8E-02 (-0.09 – 0.22)	0.04 (-0.09 – 0.26)	0.05 (-0.12 – 0.29)	0.08 (-0.16 – 0.39)
	p	3.7E-19 (1.3E-54 – 2.1E-04)	2.1E-03 (7.5E-08 – 0.12)	8.0E-08 (1.2E-19 – 0.02)	0.06 (8.0E-04 – 0.34)	2.7E-16 (3.5E-48 – 8.6E-05)	4.1E-03 (4.1E-07 – 0.22)	4.7E-08 (5.5E-20 – 0.02)	0.02 (2.1E-04 – 0.25)
**COx-a**	*r*	–	0.09 (-0.5 – 0.34)	–	-2.3E-03 (-0.38 – 0.33)	–	0.1 (-0.1 – 0.31)	–	0.04 (-0.48 – 0.39)
	p	–	0.01 (3.7E-05 – 0.21)	–	6.7E-05 (3.3E-11 – 0.1)	–	7.9E-05 (6.6E-10 – 0.09)	–	5.2E-04 (1.4E-06 – 0.03)
**250Hz Sampled Data**									
**rSO_2**	*r*	0.11 (-0.19 – 0.36)	0.14 (-0.29 – 0.44)	-0.02 (-0.15 – 0.07)	-0.07 (-0.19 – 0.09)	0.02 (-0.08 – 0.19)	0.04 (-0.1 – 0.26)	0.03 (-0.09 – 0.24)	0.12 (-0.16 – 0.41)
	p	0 (0 – 0)	1.7E-03 (6.4E-08 – 0.18)	0 (0 – 1.6E-220)	0.07 (7.2E-04 – 0.35)	0 (0 – 0)	4.1E-03 (3.5E-07 – 0.2)	0 (0 – 2.9E-61)	0.02 (2.2E-04 – 0.25)
**COx-a**	*r*	–	0.12 (-0.42 – 0.35)	–	-0.02 (-0.4 – 0.32)	–	0.08 (-0.1 – 0.32)	–	0.04 (-0.48 – 0.39)
	p	–	0.02 (2.2E-05 – 0.26)	–	6.0E-05 (2.6E-11 – 0.04)	–	1.1E-04 (9.2E-10 – 0.11)	–	5.2E-04 (1.4E-06 – 0.03)

COx-a, cerebral oximetry index with arterial blood pressure; IQR, interquartile range; MAD, median absolute deviation; r-value, Pearson correlation coefficient; rSO_2, regional cerebral oxygen saturation.

File S4c: Subgrouped Bland-Altman Analysis Between NIRS OxyMon and INVOS System Signals

Physiologic Variable	Value	Median (IQR)							
		Baseline		Neurovascular Coupling		Orthostatic Challenge		Vascular Chemo-Reactivity	
		Raw Data	10-Second Decimated Data	Raw Data	10-Second Decimated Data	Raw Data	10-Second Decimated Data	Raw Data	10-Second Decimated Data
**1Hz Sampled Data**									
rSO_2	Bias	30.53 (21.1 – 36.94)	30.53 (21 – 36.9)	33.46 (22.27 – 37.74)	33.4 (22.25 – 37.81)	31.29 (21.37 – 35.94)	31.3 (21.39 – 35.93)	27.02 (16.49 – 33.72)	26.97 (16.49 – 33.74)
	Lower LoA	24.34 (12.61 – 30.86)	25.2 (14.64 – 30.81)	27.45 (18.34 – 32.62)	28.13 (18.8 – 32.93)	25 (13.4 – 28.83)	25.41 (13.75 – 28.94)	23.4 (12.09 – 29.87)	23.37 (12.44 – 30.15)
	Upper LoA	38.18 (26.75 – 42.83)	37.81 (26.5 – 42.56)	37.71 (26.62 – 43.3)	37.63 (26.16 – 42.62)	37.08 (26.62 – 43.44)	36.59 (26.01 – 43.28)	30.99 (19.84 – 37.48)	30.57 (19.27 – 37.4)
	LoA Spread	11.25 (8.75 – 15.6)	10.49 (7.95 – 15.85)	8.31 (6.47 – 9.99)	7.64 (5.92 – 9.57)	11.35 (9.86 – 13.94)	10.59 (9.41 – 13.27)	7.32 (5.61 – 9.19)	6.59 (5.13 – 8.36)
	Relative Bias	220 (153.92 – 348.87)	234.9 (164.66 – 365.41)	354.64 (235.74 – 467.16)	378.92 (267.81 – 508.45)	243.71 (161.84 – 343.26)	260.88 (173.67 – 351.5)	366.86 (184.38 – 475.15)	393.55 (209.31 – 515.91)
	Regression Slope	1.11 (0.2 – 1.88)	1.1 (0.26 – 1.97)	1.76 (1.29 – 1.97)	1.79 (1.29 – 1.97)	1.15 (0.76 – 1.47)	1.15 (0.8 – 1.49)	1.7 (1.06 – 1.98)	1.72 (1.16 – 1.95)
	Regression Intercept	-38.5 (-76.5 – 24.01)	-36.39 (-79.53 – 20.19)	-67.28 (-76.41 – -43.08)	-67.34 (-76.79 – -42.84)	-33.25 (-52.65 – -11)	-34.85 (-53.2 – -7.69)	-63.27 (-79.05 – -28.49)	-62.62 (-81.59 – -34.37)
COx-a	Bias	–	-0.11 (-0.28 – 0.03)	–	0.02 (-0.11 – 0.2)	–	-0.11 (-0.19 – 0)	–	-0.02 (-0.11 – 0.07)
	Lower LoA	–	-0.68 (-0.9 – -0.46)	–	-0.62 (-0.79 – -0.48)	–	-0.81 (-1.12 – -0.71)	–	-0.48 (-0.67 – -0.28)
	Upper LoA	–	0.45 (0.27 – 0.85)	–	0.73 (0.51 – 0.92)	–	0.66 (0.5 – 0.85)	–	0.45 (0.33 – 0.6)
	LoA Spread	–	1.28 (0.89 – 1.57)	–	1.3 (1.08 – 1.73)	–	1.61 (1.28 – 1.85)	–	0.95 (0.65 – 1.21)
	Relative Bias	–	-12.74 (-24.97 – 3.91)	–	2.25 (-9.3 – 13.53)	–	-7.44 (-13.01 – 0.35)	–	-2.18 (-12.58 – 9.76)
	Regression Slope	–	0.18 (-0.45 – 0.92)	–	-0.02 (-0.43 – 0.36)	–	-0.07 (-0.25 – 0.18)	–	-0.24 (-0.65 – 0.24)
	Regression Intercept	–	-0.1 (-0.27 – 0.1)	–	0.03 (-0.11 – 0.23)	–	-0.11 (-0.21 – -0.02)	–	-0.02 (-0.1 – 0.11)
**250Hz Sampled Data**									
rSO_2	Bias	30.53 (21.04 – 37.13)	30.66 (20.89 – 37.05)	33.46 (22.26 – 37.74)	33.4 (22.25 – 37.81)	31.29 (21.37 – 35.94)	31.31 (21.38 – 35.93)	27.02 (16.47 – 33.72)	26.98 (16.48 – 33.79)
	Lower LoA	21.01 (10.95 – 29.54)	24.42 (12 – 30.75)	26.17 (14.53 – 32.31)	27.58 (18.78 – 32.92)	24.79 (11.93 – 28.76)	25.23 (13.79 – 28.93)	22.79 (9.77 – 29.88)	22.88 (12.41 – 30.19)
	Upper LoA	38.24 (27.94 – 44.8)	37.87 (26.61 – 42.57)	38.45 (26.88 – 43.94)	37.59 (26.16 – 42.82)	37.08 (27.46 – 43.85)	36.59 (26 – 43.23)	31.66 (20.16 – 37.8)	30.59 (19.25 – 37.45)
	LoA Spread	11.31 (9.16 – 22.39)	10.47 (8.35 – 15.94)	8.82 (6.61 – 11.11)	7.65 (5.92 – 9.57)	11.68 (10.04 – 14.65)	10.6 (9.42 – 13.28)	7.86 (5.88 – 9.78)	6.57 (5.18 – 8.23)
	Relative Bias	204.01 (115.12 – 347.85)	238.97 (163.24 – 367.81)	346.32 (193.31 – 466.99)	379.76 (254.73 – 511.56)	242.74 (149.83 – 327.37)	260.67 (174.35 – 351.4)	356.59 (161.23 – 456.35)	393.15 (199.64 – 536.77)
	Regression Slope	1.01 (-0.39 – 1.76)	1.1 (0.14 – 1.99)	1.7 (0.77 – 1.92)	1.79 (1.32 – 1.98)	1.1 (0.49 – 1.46)	1.15 (0.82 – 1.49)	1.59 (0.58 – 1.83)	1.69 (1.08 – 1.98)
	Regression Intercept	-28.06 (-61.25 – 57.9)	-36.05 (-80.89 – 26.54)	-64.44 (-73.03 – -16.27)	-67.41 (-77.77 – -43.04)	-29.25 (-49.03 – -2.42)	-35.05 (-53.17 – -6.83)	-58.15 (-70.41 – -16.02)	-62.83 (-82.91 – -29.86)
COx-a	Bias	–	-0.09 (-0.28 – 0.04)	–	0.03 (-0.11 – 0.19)	–	-0.11 (-0.2 – 0.01)	–	-0.02 (-0.13 – 0.08)
	Lower LoA	–	-0.66 (-0.91 – -0.45)	–	-0.61 (-0.79 – -0.47)	–	-0.82 (-1.11 – -0.71)	–	-0.51 (-0.63 – -0.32)
	Upper LoA	–	0.42 (0.23 – 0.84)	–	0.73 (0.51 – 0.91)	–	0.65 (0.51 – 0.86)	–	0.45 (0.31 – 0.58)
	LoA Spread	–	1.18 (0.88 – 1.53)	–	1.31 (1.09 – 1.75)	–	1.63 (1.33 – 1.85)	–	0.96 (0.65 – 1.22)
	Relative Bias	–	-11.93 (-25.46 – 4.42)	–	2.49 (-8.46 – 14.44)	–	-8.4 (-12.59 – 0.74)	–	-2.42 (-16.68 – 10.64)
	Regression Slope	–	0.14 (-0.49 – 0.83)	–	-0.1 (-0.42 – 0.37)	–	-0.06 (-0.24 – 0.22)	–	-0.27 (-0.85 – 0.3)
	Regression Intercept	–	-0.1 (-0.27 – 0.08)	–	0.04 (-0.12 – 0.24)	–	-0.12 (-0.21 – -0.01)	–	-0.02 (-0.11 – 0.11)

COx-a, cerebral oximetry index with arterial blood pressure; IQR, interquartile range; MAD, median absolute deviation; r-value, Pearson correlation coefficient; rSO_2, regional cerebral oxygen saturation.

File S4d: Subgrouped Cross-Correlation Function Best Lag Between NIRS OxyMon and INVOS System Signals

Physiologic Variable	Best Lag [Median (IQR)]							
	Baseline		Neurovascular Coupling		Orthostatic Challenge		Vascular Chemo-Reactivity	
	Raw Data	10-Second Decimated Data	Raw Data	10-Second Decimated Data	Raw Data	10-Second Decimated Data	Raw Data	10-Second Decimated Data
**1Hz Sampled Data**								
**rSO_2**	0 (0 – 0)	0 (0 – 0)	0 (0 – 0)	0 (0 – 0)	0 (0 – 0)	0 (0 – 0)	0 (0 – 0)	0 (0 – 0)
**COx-a**	–	-1 (-12 – 12)	–	7 (-15.5 – 42)	–	1.5 (-45.5 – 58.25)	–	-0.5 (-20.75 – 7.25)
**250Hz Sampled Data**								
**rSO_2**	0 (0 – 0)	0 (0 – 0)	0 (0 – 0)	0 (0 – 0)	0 (0 – 0)	0 (0 – 0)	0 (0 – 0)	0 (0 – 0)
**COx-a**	–	0 (-9.5 – 13.75)	–	7 (-7.25 – 44.25)	–	0 (-64 – 38.25)	–	-7 (-25.5 – 1.75)

COx-a, cerebral oximetry index with arterial blood pressure; IQR, interquartile range; rSO_2, regional cerebral oxygen saturation.

File S4e: Subgrouped Median and IQR of Optimal ARIMA Models Based on AIC

Physiologic Variable	Optimal ARIMA Models (Median [IQR])							
	Baseline		Neurovascular Coupling		Orthostatic Challenge		Vascular Chemo-Reactivity	
	1Hz	250Hz	1Hz	250Hz	1Hz	250Hz	1Hz	250Hz
**ABP**	(1,1,4) [(1,1,1) – (4,1,0)]	(1,1,8) [(1,1,1) – (3,1,2)]	(4,1,3) [(2,1,5) – (5,1,7)]	(4,1,3) [(2,1,5) – (7,1,0)]	(4,1,3) [(2,1,4) – (6,1,8)]	(3,1,4) [(2,1,3) – (5,1,3)]	(2,1,2) [(1,1,1) – (3,1,2)]	(2,1,3) [(1,1,5) – (4,1,1)]
**rSO_2_Invos**	(1,1,4) [(1,1,1) – (2,1,3)]	(1,1,1) [(1,1,1) – (2,1,2)]	(2,1,6) [(2,1,0) – (4,1,4)]	(2,1,7) [(2,1,2) – (5,1,5)]	(4,1,3) [(3,1,2) – (6,1,5)]	(4,1,4) [(3,1,2) – (5,1,10)]	(2,1,1) [(1,1,7) – (3,1,1)]	(2,1,2) [(1,1,4) – (3,1,2)]
**COx-a_Invos**	(1,1,1) [(1,1,0) – (2,1,2)]	(1,1,1) [(1,1,0) – (2,1,2)]	(2,1,0) [(1,1,0) – (3,1,2)]	(1,1,7) [(1,1,0) – (2,1,3)]	(2,1,2) [(1,1,1) – (4,1,9)]	(3,1,1) [(1,1,3) – (5,1,3)]	(1,1,4) [(1,1,0) – (2,1,3)]	(1,1,5) [(1,1,0) – (2,1,2)]
**rSO_2_OxyMon**	(1,1,3) [(1,1,1) – (2,1,3)]	(1,1,7) [(1,1,1) – (2,1,3)]	(2,1,1) [(2,1,0) – (3,1,3)]	(2,1,1) [(1,1,6) – (4,1,0)]	(3,1,3) [(1,1,2) – (4,1,3)]	(2,1,3) [(1,1,3) – (3,1,6)]	(1,1,2) [(1,1,1) – (2,1,3)]	(1,1,5) [(1,1,1) – (2,1,3)]
**COx-a_OxyMon**	(1,1,3) [(1,1,0) – (3,1,1)]	(1,1,2) [(1,1,0) – (3,1,0)]	(1,1,3) [(1,1,0) – (2,1,2)]	(1,1,3) [(1,1,0) – (2,1,2)]	(2,1,3) [(1,1,5) – (3,1,10)]	(2,1,3) [(1,1,3) – (3,1,4)]	(1,1,4) [(1,1,0) – (2,1,2)]	(1,1,1) [(1,1,0) – (2,1,2)]

ABP, arterial blood pressure; AIC, Akaike Information Criterion; ARIMA, autoregressive integrative moving average; COx-a, cerebral oximetry index with ABP; IQR, interquartile range; rSO_2, regional cerebral oxygen saturation.

File S4f: Subgrouped Signal Responsiveness using Impulse Response Coefficients of Optimal VARIMA model

Direction	% (count)							
	Baseline		Neurovascular Coupling		Orthostatic Challenge		Vascular Chemo-Reactivity	
	>0.1%	NA	>0.1%	NA	>0.1%	NA	>0.1%	NA
**1Hz Sampled Data**								
**ABP → rSO_2_Invos**	64% (32)	4% (2)	84% (42)	4% (2)	96% (48)	0% (0)	84% (42)	2% (1)
**rSO_2_Invos → ABP**	66% (33)	4% (2)	84% (42)	4% (2)	96% (48)	0% (0)	86% (43)	2% (1)
**ABP → rSO_2_OxyMon**	68% (34)	6% (3)	88% (44)	2% (1)	84% (42)	6% (3)	64% (32)	2% (1)
**rSO_2_OxyMon → ABP**	70% (35)	6% (3)	92% (46)	2% (1)	88% (44)	6% (3)	68% (34)	2% (1)
**250Hz Sampled Data**								
**ABP → rSO_2_Invos**	68% (34)	6% (3)	86% (43)	4% (2)	94% (47)	0% (0)	92% (46)	4% (2)
**rSO_2_Invos → ABP**	64% (32)	6% (3)	84% (42)	4% (2)	92% (46)	0% (0)	92% (46)	4% (2)
**ABP → rSO_2_OxyMon**	78% (39)	4% (2)	88% (44)	0% (0)	86% (43)	0% (0)	74% (37)	4% (2)
**rSO_2_OxyMon → ABP**	82% (41)	4% (2)	94% (47)	0% (0)	88% (44)	0% (0)	80% (40)	4% (2)

ABP, arterial blood pressure; p-order, autoregressive order; rSO_2, regional cerebral oxygen saturation; VARIMA, vector autoregressive integrative moving average.

File S4g: Subgrouped Granger Causal Directionality Results Based on Greater F-Statistic

Signal	Direction	[% (count)]			
		Baseline	Neurovascular Coupling	Orthostatic Challenge	Vascular Chemo-Reactivity
**1Hz Sampled Data**					
**ABP & rSO_2_Invos**	ABP → rSO_2_Invos	54% (27)	54% (27)	44% (22)	50% (25)
	rSO_2_Invos → ABP	46% (23)	46% (23)	56% (28)	50% (25)
	NA	6% (3)	2% (1)	0% (0)	2% (1)
**ABP & rSO_2_OxyMon**	ABP → rSO_2_OxyMon	42% (21)	48% (24)	56% (28)	52% (26)
	rSO_2_OxyMon → ABP	58% (29)	52% (26)	44% (22)	48% (24)
	NA	4% (2)	4% (2)	0% (0)	2% (1)
**250Hz Sampled Data**					
**ABP & rSO_2_Invos**	ABP → rSO_2_Invos	48% (24)	42% (21)	48% (24)	38% (19)
	rSO_2_Invos → ABP	52% (26)	58% (29)	52% (26)	62% (31)
	NA	4% (2)	0% (0)	0% (0)	4% (2)
**ABP & rSO_2_OxyMon**	ABP → rSO_2_OxyMon	52% (26)	46% (23)	62% (31)	56% (28)
	rSO_2_OxyMon → ABP	48% (24)	54% (27)	38% (19)	44% (22)
	NA	6% (3)	4% (2)	0% (0)	4% (2)

ABP, arterial blood pressure; p-order, autoregressive order; rSO_2, regional cerebral oxygen saturation.
